# Enhancing C_2+_ product selectivity in CO_2_ electroreduction by enriching intermediates over carbon-based nanoreactors†

**DOI:** 10.1039/d4sc01735h

**Published:** 2024-05-01

**Authors:** Min Wang, Chunjun Chen, Shuaiqiang Jia, Shitao Han, Xue Dong, Dawei Zhou, Ting Yao, Minghui Fang, Mingyuan He, Wei Xia, Haihong Wu, Buxing Han

**Affiliations:** a Shanghai Key Laboratory of Green Chemistry and Chemical Processes, State Key Laboratory of Petroleum Molecular & Process Engineering, School of Chemistry and Molecular Engineering, East China Normal University Shanghai 200062 China wxia@chem.ecnu.edu.cn hhwu@chem.ecnu.edu.cn hanbx@iccas.ac.cn; b Institute of Eco-Chongming 20 Cuiniao Road, ChenjiaTown, Chongming District Shanghai 202162 China; c Beijing National Laboratory for Molecular Sciences, CAS Key Laboratory of Colloid and Interface and Thermodynamics, CAS Research/Education Center for Excellence in Molecular Sciences, Institute of Chemistry, Chinese Academy of Sciences Beijing 100190 China

## Abstract

Electrochemical CO_2_ reduction reaction (CO_2_RR) to multicarbon (C_2+_) products faces challenges of unsatisfactory selectivity and stability. Guided by finite element method (FEM) simulation, a nanoreactor with cavity structure can facilitate C–C coupling by enriching *CO intermediates, thus enhancing the selectivity of C_2+_ products. We designed a stable carbon-based nanoreactor with cavity structure and Cu active sites. The unique geometric structure endows the carbon-based nanoreactor with a remarkable C_2+_ product faradaic efficiency (80.5%) and C_2+_-to-C_1_ selectivity (8.1) during the CO_2_ electroreduction. Furthermore, it shows that the carbon shell could efficiently stabilize and highly disperse the Cu active sites for above 20 hours of testing. A remarkable C_2+_ partial current density of−323 mA cm^−2^ was also achieved in a flow cell device. *In situ* Raman spectra and density functional theory (DFT) calculation studies validated that the *CO_atop_ intermediates are concentrated in the nanoreactor, which reduces the free energy of C–C coupling. This work unveiled a simple catalyst design strategy that would be applied to improve C_2+_ product selectivity and stability by rationalizing the geometric structures and components of catalysts.

## Abstract 2

A carbon-based nanoreactor could enrich *CO intermediates through steric confinement, thus facilitating electrocatalytic C–C coupling.

## Graphical abstract

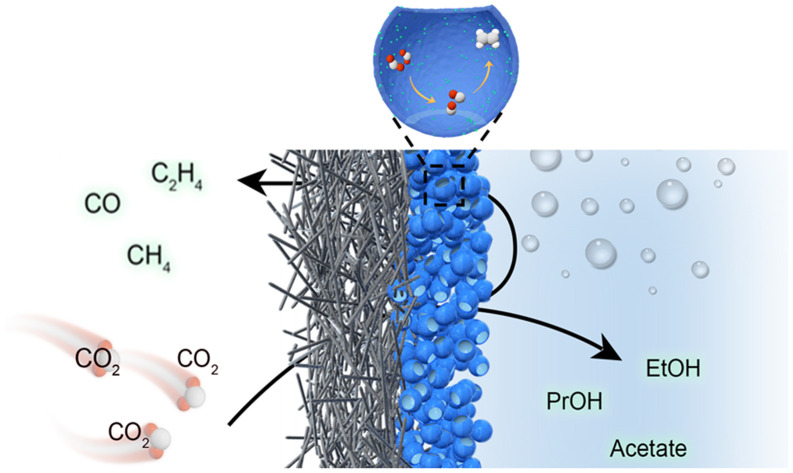

## Introduction

The escalating emission of greenhouse gases exacerbates climate change, posing a greater threat to the future of humanity. Carbon dioxide (CO_2_), one of the primary greenhouse gases, plays a crucial role in this process. Consequently, the electrocatalytic reduction of CO_2_ into valuable chemicals and fuels holds immense significance in mitigating climate change, promoting a closed carbon cycle, and facilitating the storage of renewable electricity.^1–3^ The primary outputs of the electrochemical CO_2_ reduction reaction (CO_2_RR) consist of C_1_ products and multicarbon (C_2+_) products. Within these products, C_2+_ products hold greater economic significance within the chemical industry due to their higher commercial value.^4–6^ Currently, extensive efforts have been devoted to developing catalysts to obtain high-productivity of C_2+_ products.^7–10^ Among them, copper (Cu) is recognized as the unique metal to efficaciously break the stable CO_2_ bonds and form C–C bonds, a key step towards C_2+_ products.^11–14^ However, the challenge of C_2+_ selectivity still limits its economic competitiveness, and more advanced strategies are needed to design high-performance catalysts.

Based on previous studies, C–C coupling is considered a crucial route for the formation of C_2+_ products in the CO_2_RR, whereas the C–C coupling reaction relies heavily on the *CO intermediate dimerization.^15^ Achieving a local high concentration of *CO intermediates at the active sites is crucial for initiating the dimerization process between neighbouring *CO intermediates.^16^ But *CO species tended to diffuse from the catalyst surface to the electrolyte, and it may result in their premature discharge from the active sites.^17^ This premature release severely hampers the efficiency of C–C coupling, thus reducing the overall performance of the CO_2_RR process.^18^ A simple way to increase the local concentration of *CO species is to increase the diffusion resistance and extend the diffusion path length.

Previous studies have demonstrated that the confinement effect can alter the diffusion kinetics and effectively improve the local concentration of key intermediates.^19^ Sargent *et al.* applied finite element method (FEM) simulations and experimental analyses to explore the C–C coupling reactions by the confinement effect.^20^ They demonstrated that Cu_2_O with the cavity structure led to a high surface coverage of intermediates and improved the electrocatalytic conversion of CO-to-propanol. Yu *et al.* conducted a comprehensive investigation on the multicavities of Cu_2_O *via* intermediate confinement to enhance the selectivity of CO_2_ electroreduction to C_2+_ fuels.^21^ These findings revealed that this unique cavity structure effectively suppressed the loss of key intermediates, and enhanced their local concentration at active sites, thus increasing the chance of C–C coupling reactions. Studies on the steric confinement effect in the CO_2_RR have substantiated its capability to enhance the selectivity of multicarbon products.^22,23^ However, a persistent challenge in current studies about Cu-based nanoreactors is Cu surface reconstruction and compositional changes during electrolysis, which makes product selectivity and activity decline significantly.^24^ Also, the designability of pure Cu-based catalysts is very limited.

It is known that supported catalysts, in which active sites are anchored on supports, have some obvious advantages, such as the size and dispersion of the active sites can be tuned, and the synergy of the active sites and supports.^25^ Specifically, carbon materials exhibit advantages in electrocatalysis, including strong stability, ease of morphological adjustments and optimized reaction intermediate adsorption that make it an excellent candidate for supporting active sites.^26^ Xia *et al.* proposed a carbon protected indium oxide electrocatalyst, where the carbon layer not only prevents the reductive corrosion of indium oxide during electrolysis, but also optimizes the intermediate adsorption, thus improving the stability and activity of CO_2_ reduction.^27^ Therefore, it is important to introduce a carbon layer into catalyst design to achieve electrochemical stability while maintaining high activity.

Combining steric confined carbon supported nanoreactors with highly active Cu sites would establish a distinctive class of catalysts with both exceptional C_2+_ product selectivity and stability. Thus, we began using FEM simulations to explore the influence of cavity structures on the accumulation of *CO intermediates and C–C coupling. Guided by FEM results, we synthesized a Cu/C-cavity nanoreactor in which Cu species are supported in the carbon shell. The unique structure endows the Cu/C-cavity catalyst with high CO_2_ electrocatalytic performance. As a result, the nanoreactor achieved a remarkable multicarbon (C_2+_) product faradaic efficiency (FE) of 80.5% and a higher C_2+_-to-C_1_ ratio of 8.1 at −1.2 V *vs.* RHE. Based on the highly dispersed Cu active sites on the carbon support to prevent agglomeration, the nanoreactor performed continuous electrolysis for more than 20 hours. *In situ* Raman spectra and density functional theory (DFT) calculation results revealed that the cavity of the nanoreactor could concentrate *CO_atop_ intermediates and reduce their dimerization barriers. This work introduces a convenient and efficient synthesis strategy to enhance the selectivity of multicarbon products and stability for the electrochemical CO_2_RR. This strategy could applicable to other reactions, rationalizing the morphology and active components of the catalyst, and concentrating key intermediates through nanoconfinement to improve the selectivity of ideal products.

## Results and discussion

**Fig. 1:**
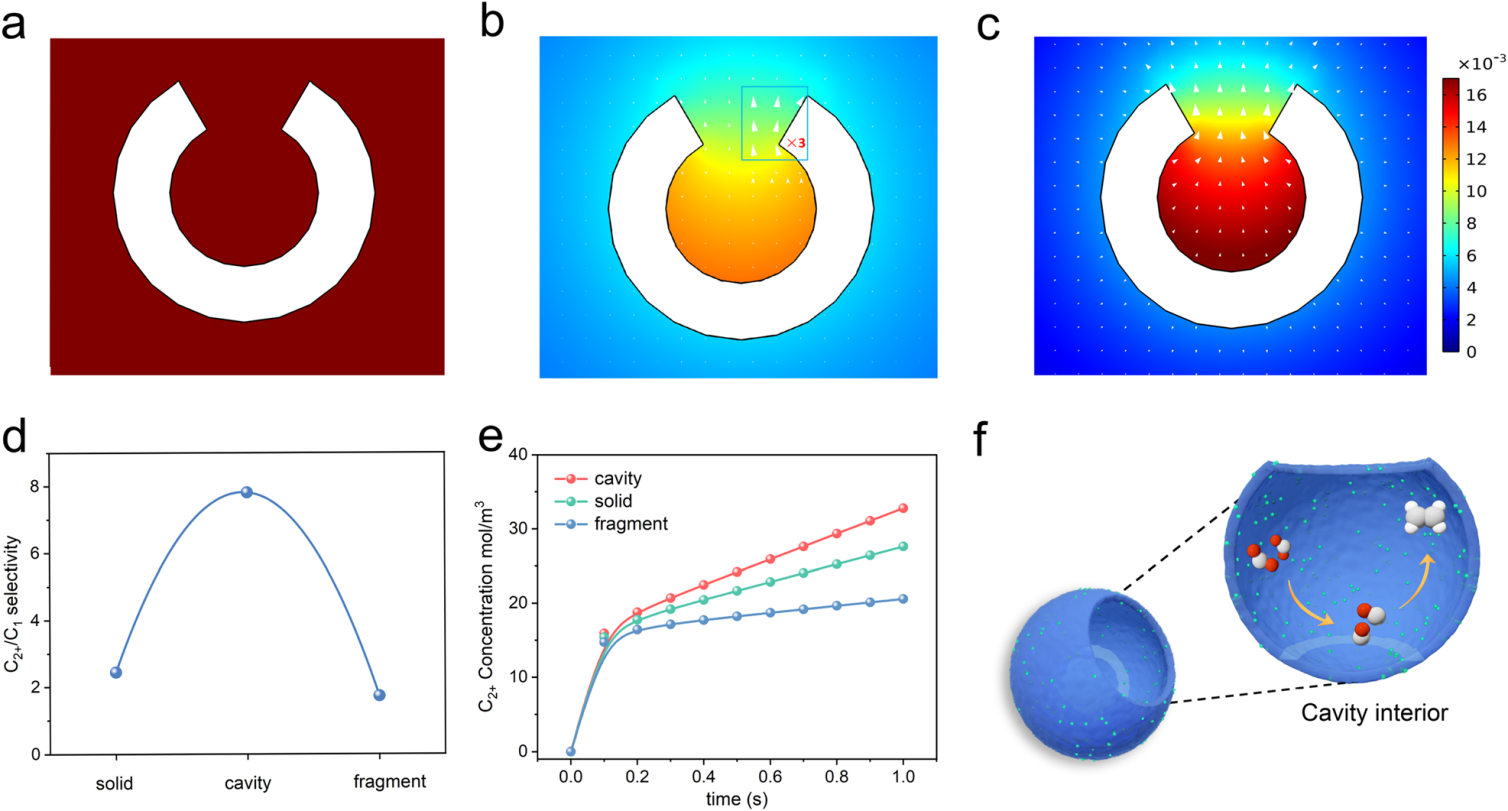
Computed concentration and flux distribution of species. (a) CO_2_, (b) *CO, and (c) C_2+_ concentrations (color scale, in mol L^−1^) and flux distributions (arrows) on the cavity structure. (d) Simulation results of the C_2+_/C_1_ product selectivity on the cavity, solid and fragment structure. (e) Simulation results of the time-dependent variation of C_2+_ concentration on cavity, solid and fragment structure. (f) The diagram displays how the cavity confinement effect promotes *CO intermediate dimerization and transformation to C_2_H_4_. Red, oxygen; grey, carbon; white, hydrogen.

It is assumed that the cavity structure nanoreactor could retard the diffusion kinetics through the confinement effect and concentrate *CO species, thus increasing the possibility for C–C coupling. To verify the hypothesis, we applied FEM simulations to explore the prospects of cavity structure in enhancing C_2+_ product selectivity. A hollow spherical shell model with circular openings (with an outer diameter of 39 nm and an inner diameter of 6.5 nm) was used to represent the nanoreactor immersed in an electrolyte (see details in the ESI†). The out flux of *CO and C_2_ products on the inner and outer surfaces of the nanoreactor was monitored. Results of the simulation indicated that CO_2_ molecules first diffused to the surface (Fig. 1a). Then, CO_2_ was adsorbed and reduced into *CO at the interior and outer surfaces of the spherical shell (Fig. 1b). Finally, the *CO species could desorb from the surface as C_1_ products (CO assumed) or be coupled with other *CO to form C_2_ products (C_2_H_4_ assumed). The surface C_2_ species could diffuse into the electrolyte or continue to react with another *CO to produce C_3_ products. Consistent with our hypothesis, the cavity structure could restrict the diffusion of internally generated *CO intermediates to the outside of the cavity (Fig. 1b, arrows), leading to the accumulation of *CO species inside the cavity and the concentration of the *CO species was significantly increased (Fig. 1b, colour map). The results lead to a high concentration of *CO intermediates required for the formation of C_2_ products, and facilitated the conversion to C_2_ products (Fig. 1c).

Furthermore, the microenvironments over the interface of the fully closed and fully open structures were also monitored by FEM. The solid and fragment models were used to represent the fully closed and fully open structure catalyst immersed in an electrolyte. As *CO species tended to escape from the catalyst surface to the electrolyte, both the solid and fragment structures cannot prevent the diffusion of *CO from its surface to the electrolyte (Fig. S1a–f†). Therefore, these two structures were ineffective in enhancing the coverage of *CO species at active sites, resulting in no enhancement in the yield of C_2+_ products. The solid and fragment structures exhibited limitations in promoting C–C coupling. Fig. 1d shows coincident simulating results of the C_2+_/C_1_ ratios of the three structures. The C_2+_-to-C_1_ simulated ratios of cavity, solid and fragment structures were 7.81, 2.44, 1.73, respectively.

We also simulated the time-dependent variation of C_2+_ concentration on the cavity, solid and fragment structure models, and the results are shown in Fig. 1e. The C_2+_ concentration on the three models kept increasing with time, and the cavity model was obviously larger than that of the solid and fragment structure models at the same time. This distinction arose from the cavity structure, which significantly slowed down the diffusion kinetics, and improved the local coverage of *CO, thus facilitating the formation of C_2+_ products. In contrast, the diffusion of the *CO on solid and fragment structures cannot be retarded. These simulation results showed that cavity structures could promote the formation of C_2+_ products through steric nanoconfinement effects (Fig. 1f).

**Fig. 2:**
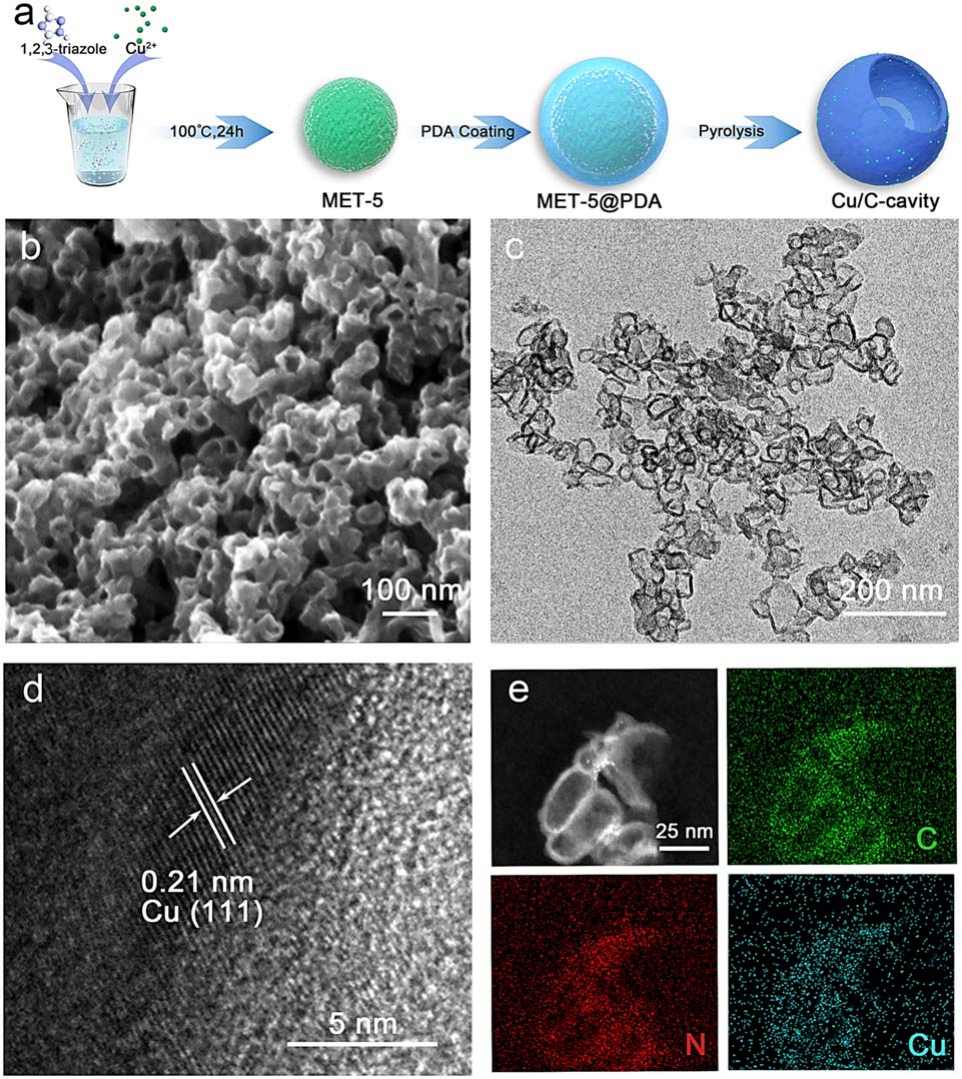
Morphology characterization of the Cu/C-cavity catalyst. (a) The synthesis process of the Cu/C-cavity catalyst. Cu/C-cavity catalyst imaged by (b) SEM, (c) TEM, and (d) HRTEM. (e) High-angle annular dark field (HAADF) and mapping images of the Cu/C-cavity catalyst, showing the homogeneous distribution of C (green), N (red), and Cu (blue).

Under the guidance of FEM simulation results, based on the synthesis mechanism of the self-template method,^28^ the carbon-based nanoreactor (Cu/C-cavity) was synthesized. The synthetic protocol of the Cu/C-cavity catalyst is illustrated in Fig. 2a. First, the template MET-5 was synthesized by a solvothermal method^29^ (Fig. S2†), and then coated with a polydopamine (PDA) outer layer to obtain MET-5@PDA (Fig. S3†). Thermogravimetric analysis of MET-5 and MET-5@PDA was carried out (Fig. S4†). The removal rate of template MET-5 could be regulated by controlling the calcination time and heating rate, which results in different structures,^30^ and the Cu/C-cavity and Cu/C-fragment (fully open structures) catalysts could be obtained. The Cu/C-solid (fully closed structures) was obtained by carbonizing MET-5.

The scanning electron microscopy (SEM) images in Fig. 2b and S5† show the overall morphology of the Cu/C-cavity catalyst. Spherical particles with cavities were observed. In addition, transmission electron microscopy (TEM) images in Fig. 2c, S6 and S7† revealed the hollow morphology and open structures. The high-resolution TEM (HRTEM) image of the Cu/C-cavity catalyst displayed the lattice fringe of the crystal plane of Cu (111) (Fig. 2d). In addition, the SEM and TEM images confirmed the synthesis of Cu/C-solid and Cu/C-fragment catalysts (Fig. S8–S11†). The BET surface areas of Cu/C-cavity, Cu/C-solid, and Cu/C-fragment are shown in Fig. S12.† The high-angle annular dark field (HAADF) image in Fig. 2e further confirms the hollow spherical structure formation of the Cu/C-cavity catalyst. The energy-dispersive X-ray spectroscopy (EDX) elemental mapping images showed uniform distribution of C, N, and Cu in the Cu/C-cavity catalyst.

**Fig. 3:**
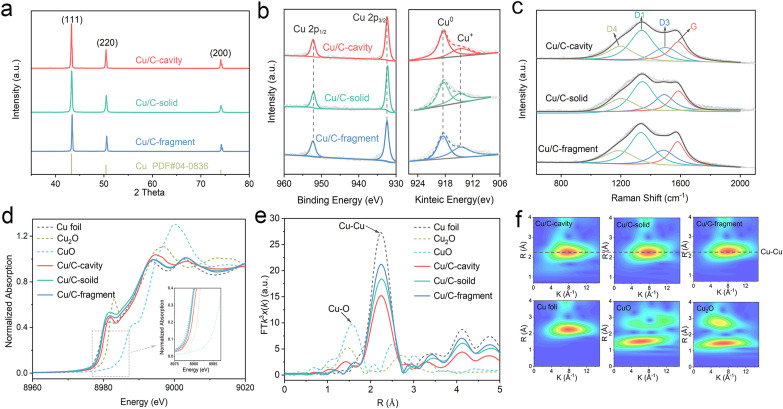
Chemical structural characterization of different catalysts. (a) XRD, (b) XPS spectra of Cu 2p and Cu LMM Auger, (c) Raman spectra, (d) Cu K-edge XANES, (e) Fourier transformed Cu K-edge EXAFS spectra and (f) Morlet WT of the k3-weighted EXAFS of Cu/C-cavity, Cu/C-solid and Cu/C-fragment with the references.

The chemical states and composition of the Cu/C-cavity, Cu/C-solid and Cu/C-fragment catalysts were confirmed by a series of techniques. The powder X-ray diffraction (XRD) patterns results are presented in Fig. 3a, and the diffraction patterns at 43.2, 50.4, and 74.1 could be indexed to the Cu (111), Cu (200), and Cu (220) planes, respectively (PDF #04-0836). The XPS survey spectra illustrated the existence of Cu, O, N, and C elements in the three materials (Fig. S13a†). The Cu 2p_3/2_ peak and Cu 2p_1/2_ peak in the XPS spectra were observed at 932.3 eV and 952.2 eV (Fig. 3b), respectively. The Cu LMM Auger verified that the Cu species in the Cu/C-cavity, Cu/C-solid, and Cu/C-fragment catalysts existed in the form of Cu^0^ (918.3 eV, predominant) and Cu^+^ (914.7 eV). The minor amount of Cu^+^ may result from the partial oxidation of Cu during characterization. In addition, the N 1s peak could be deconvolved into pyridinic-N (398.48 eV), graphitic-N (401.4 eV), and oxidic-N (402.35 eV) peaks (Fig. S13b†).^31^ The C 1s spectrum could be fitted into three peaks, corresponding to C–C (284.6 eV), C–N (285.9 eV), and C–O (287.2 eV) (Fig. S13c†).^32^ The O 1s spectrum could be deconvolved into O–C (530.8 eV), O=C (532.1 eV), and O=C–O (533.5 eV) (Fig. S13d†).^33^

The structures of Cu/C-cavity, Cu/C-solid, and Cu/C-fragment catalysts were further evaluated by Raman analysis (Fig. 3c). The Raman spectra could be deconvoluted into four peaks by Gaussian–Lorentzian numerical simulation,^34^ which were the graphene edges for the D1-band (*ca.* 1360 cm^−1^), topological defects for the D3-band (*ca.* 1500 cm^−1^), polyenes for the D4-band (*ca.* 1180 cm^−1^) and graphitic lattice for the G-band (*ca.* 1580 cm^−1^).^35^ The graphitization degree of carbon was inferred by the ratio of integrated areas of the D1 and G.^36^ The calculated *I*_D1_/*I*_G_ ratios of the Cu/C-cavity, Cu/C-solid, and Cu/C-fragment catalysts were 1.65, 2.42, and 1.78, respectively, indicating that the Cu/C-cavity had a higher graphitization degree than Cu/C-solid and Cu/C-fragment catalysts.

The electronic information of the Cu/C-cavity, Cu/C-solid and Cu/C-fragment was further investigated by X-ray absorption spectroscopy (XAS) measurements. The Cu K-edge XANES spectra of the Cu/C-cavity, Cu/C-solid and Cu/C-fragment together with the references of Cu_2_O, CuO, and Cu foil are given in Fig. 3d. The X-ray absorption near edge structure (XANES) spectra (Fig. S14 and S15†) of Cu/C-cavity, Cu/C-solid and Cu/C-fragment were between the Cu foil and Cu_2_O, which proved that the Cu species was in the intermediate valence state between 0 and +1.^37^ The extended X-ray absorption fine structure (EXAFS) spectra (Fig. 3e) of Cu/C-cavity, Cu/C-solid and Cu/C-fragment displayed a main peak at 2.24 Å, corresponding to the metal Cu–Cu bond.^38^ As shown in Fig. 3f, the Morlet Wavelet Transform (WT) of the k3-weighted extended X-ray absorption fine structure (EXAFS) further proved the existence of the Cu–Cu bond in the Cu/C-cavity, Cu/C-solid and Cu/C-fragment catalysts.^39^

**Fig. 4:**
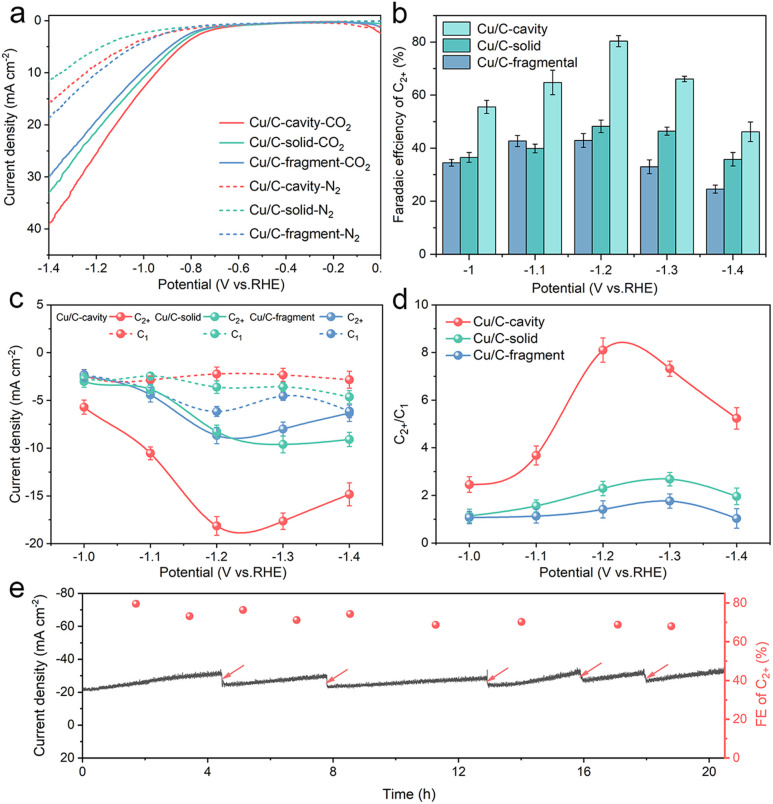
CO_2_ electrochemical reduction performance. (a) LSV curves on Cu/C-cavity, Cu/C-solid and Cu/C-fragment in CO_2_-saturated and N_2_-saturated 0.1 M CsI aqueous electrolyte. (b) C_2+_ FE from −1.0 V to −1.4 V *vs.* RHE. (c) C_2+_ and C_1_ partial current densities at −1.0 V to −1.4 V *vs.* RHE in CO_2_ saturated 0.1 M CsI aqueous electrolyte. (d) C_2+_/C_1_ products selectivity ratio from −1.0 V to −1.4 V *vs.* RHE in CO_2_ saturated 0.1 M CsI aqueous electrolyte. (e) Stability test of Cu/C-cavity at −1.2 V *vs.* RHE in CO_2_ saturated 0.1 M CsI aqueous electrolyte, the arrows indicate the renewal of the electrolyte.

The CO_2_ electroreduction activity over the Cu/C-cavity, Cu/C-solid, and Cu/C-fragment catalysts was first investigated in 0.1 M CsI aqueous solution using a typical H type cell (Fig. S16†). As shown in Fig. 4a, the linear sweep voltammetry (LSV) curves over the Cu/C-cavity, Cu/C-solid, and Cu/C-fragment catalysts exhibited a higher reduction current density in the CO_2_-saturated electrolyte than the N_2_-saturated electrolyte, demonstrating their CO_2_RR activity.^40^ Comparing these three catalysts, it was found that the Cu/C-cavity exhibited the highest reduction current density and the most positive onset potential compared to Cu/C-solid, and Cu/C-fragment catalysts. This indicated that the Cu/C-cavity achieved a higher CO_2_RR activity than the other two catalysts. The electrochemically active surface areas (ECSA) of the Cu/C-cavity, Cu/C-solid and Cu/C-fragment catalysts were obtained from the double-layer capacitance (*C*_dl_). In Fig. S17,† the Cu/C-cavity catalyst has the largest ECSA, which demonstrates that the Cu/C-cavity catalyst has the highest CO_2_RR activity.^41^ Moreover, the Nyquist plots were recorded at the open-circuit potential to investigate the reaction kinetics of electrochemical processes. The electrochemical impedance spectroscopy (EIS) (Fig. S18†) shows that the Cu/C-cavity displayed the smallest Nyquist semicircle diameter compared to Cu/C-solid and Cu/C-fragment, suggesting a much faster interfacial charge-transfer kinetics.^42^ The local high ion concentration inside the cavity structure or the higher graphitization degree of Cu/C-cavity may improve the electrical conductivity, thus accelerating charge-transfer.^43^

According to the results in Fig. S19a,† the Cu/C-cavity catalyst demonstrated the production of C_2+_ products within the potential range of −1.0 V to −1.4 V *vs.* RHE. The results showed that the FE of C_2+_ products over the Cu/C-cavity catalyst could reach up to 80.5% (1446.17 ppm) at −1.2 V *vs.* RHE (Fig. 4b). This efficiency comprised 52.2% C_2_H_4_, 18.8% C_2_H_5_OH, 5.4% CH_3_COOH, and 4.2% *n*-PrOH (Fig. S20†). Fig. S21† shows the liquid products in D_2_O (DMSO and phenol as an internal standard). Comparatively, the Cu/C-fragment and Cu/C-solid catalysts tended to produce CO-dominated C_1_ products (Fig. 4b, S19b, c and S20†). The FE over C_2+_ products on Cu/C-cavity was much higher than that over the Cu/C-solid and Cu/C-fragment catalysts. The above experimental results proved that the steric confinement effect of the cavity structure could promote C_2+_ product selectivity. The partial current densities of C_2+_ and C_1_ products of Cu/C-cavity, Cu/C-solid, and Cu/C-fragment catalysts were compared at different applied potentials. As depicted in Fig. 4c, it was evident that the Cu/C-cavity catalyst exhibited the highest C_2+_ partial current density, reaching 18.14 mA cm^−2^ at −1.2 V *vs.* RHE. In contrast, the Cu/C-solid and Cu/C-fragment showed lower maximum C_2+_ partial current densities of only 8.68 and 8.25 mA cm^−2^, respectively. The *j*_C_2+__ normalised to ECSA and Cu content of Cu/C-cavity was larger than that of the Cu/C-solid and Cu/C-fragment catalysts (Tables S1 and S2†), indicating that the high C_2+_ products selectivity over the Cu/C-cavity resulted from its unique structure.

Fig. 4d discusses the ratios of C_2+_ to C_1_ products over the Cu/C-cavity, Cu/C-solid, and Cu/C-fragment catalysts. The Cu/C-cavity exhibited higher selectivity towards C_2+_ products across all tested potentials. Particularly at −1.2 V *vs.* RHE, the Cu/C-cavity catalyst achieved maximum selectivity for C_2+_ products, with a C_2+_/C1 ratio of approximately 8.1. The ratios over the Cu/C-solid and Cu/C-fragment catalysts were 2.7 and 1.8, respectively. To further confirm the universality of our Cu/C-cavity structure, we investigated the electrochemical CO_2_RR performance of Cu/C-cavity, Cu/C-solid, and Cu/C-fragment in 0.1 M KHCO_3_ solution using a typical H type cell (Fig. S22†), and the FE of C_2+_ products over the Cu/C-cavity catalyst could reach up to 61.2% at −1.2 V *vs.* RHE, with a C_2+_/C_1_ selectivity ratio of approximately 6.14. The ratios of the Cu/C-solid and Cu/C-fragment catalysts were 2.06 and 1.31, respectively. The Cu/C-cavity also exhibited higher C_2+_ selectivity than Cu/C-solid and Cu/C-fragment in 0.1 M KHCO_3_ solution. The experimental and simulated results showed excellent agreement, and it is also proved that the cavity structure is beneficial to improve the selectivity of C_2+_ products.

Furthermore, the electrochemical CO_2_RR performances of Cu/C-cavity, Cu/C-solid, and Cu/C-fragment were studied in 1 M KOH using a flow cell within the potential range of −0.8 V to −1.2 V *vs.* RHE (Fig. S23†). The Cu/C-cavity catalyst exhibited much higher selectivity for C_2+_ products (Fig. S24†), and the FE of C_2+_ could reach up to 75.2% (6990.76 ppm) with a C_2+_/C_1_ ratio of 3.57 at −1.0 V *vs.* RHE. The *j*_C_2+__ could reach −323 mA cm^−2^, which is higher than those of most electrodes and reached industrial levels. In addition, we also conducted the stability test in a flow cell, which indicated that the stability of the electrode was satisfactory (Fig. S24e†). Fig. S25† shows the liquid products over Cu/C-cavity in the flow cell. However, the Cu/C-solid and Cu/C-fragment tended to produce CH_4_-dominated C_1_ products. The ratios of C_2+_/C_1_ over the Cu/C-solid and Cu/C-fragment catalysts were 1.15 and 1.34, respectively. The Cu/C-cavity also exhibited much higher C_2+_ selectivity than Cu/C-solid and Cu/C-fragment in the flow cell. Consequently, the above results proved that the confined geometric structure facilitates C–C coupling and the formation of multi-carbon compounds, which aligned with our previous hypothesis.

The stability of catalysts is a crucial parameter for the CO_2_RR. To assess the stability of the Cu/C-cavity catalyst, CO_2_ electrolysis was carried out at −1.2 V *vs.* RHE. As in Fig. 4e, the current density and FE did not change obviously over a period of 20 hours. The Cu/C-cavity nanoreactor was characterized by XRD and XPS techniques (Fig. S26†) after electrocatalysis showed no apparent change in the chemical state. The Cu K-edge spectra indicated that the Cu/C-cavity nanoreactor after electrocatalysis retained the characteristic feature of Cu(0), as can be further confirmed by the Cu–Cu coordination at 2.23 Å (Fig. S27†). From the TEM images (Fig. S28†) of the Cu/C-cavity after the CO_2_RR, the nanocavity structure basically remained intact, and no obvious agglomeration of particles was found. We conducted a comparative analysis of our carbon-based nanoreactor materials with other reported nanoconfinement reactors in the literature for CO_2_ electroreduction. The result proved that carbon-based nanoreactors have superior stability over other reported pure Cu-based nanoreactors (Table S3†). The superior stability is mainly from the Cu and C structure of the catalyst, and the carbon carrier effectively stabilizes the active site of Cu and protects it from the reaction environment.

**Fig. 5:**
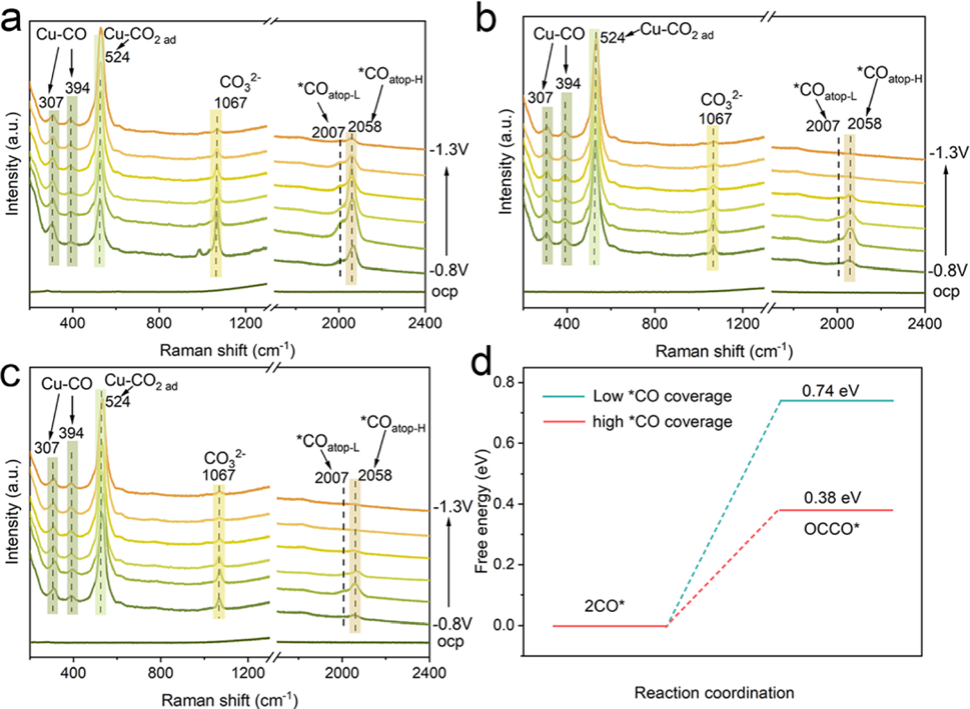
Mechanistic studies. *In situ* Raman spectra of the (a) Cu/C-cavity, (b) Cu/C-solid, and (c) Cu/C-fragment catalysts during the CO2RR under different applied potentials. (d) The free energy of the *CO dimerization step at low (blue) and high *CO coverage (red) on Cu(111).

To further explore the effect of nanoconfinement on the CO_2_RR, *in situ* Raman spectroscopy was conducted to monitor the key intermediates *CO of surface adsorption. Fig. 5a shows the *in situ* Raman spectra using the Cu/C-cavity catalyst during the CO_2_RR under different applied potentials in 0.5 M KHCO_3_ solution. The bands at 307 and 394 cm^−1^ were attributed to the CO frustrated rotation and Cu–CO vibration stretching, respectively, indicating the adsorption of *CO.^44^ The band at 524 cm^−1^ was ascribed to the adsorption of preliminary intermediates (such as CO_2_ ad) on the active sites.^45^ The band at 984 cm^−1^ was assigned to the *COO. Meanwhile, a much stronger band at 1067 cm^−1^, corresponding to carbonate could be observed. The adsorption bands in the range of 2007–2058 cm^−1^ were attributed to the atop-bound *CO (*CO_atop_), which is a key intermediate of the CO–CO coupling.^46^ The *CO_atop_ peak could split into two bands. The low frequency band (LFB) at 2007 cm^−1is^ ascribed to *CO_atop_ on the terrace (*CO_atop_–L), and the high frequency band (HFB) at 2058 cm^−1^ is associated with *CO_atop_ on the low coordinated sites (*CO_atop_–H). The *CO_atop_ peak intensity increased and then decreased with the potential scanned, indicating that *CO intermediates were accumulated and then consumed.

Furthermore, the *in situ* Raman spectra of Cu/C-solid and Cu/C-fragment were also recorded (Fig. 5b and c). The intensities of the peaks for linearly adsorbed *CO_atop_ on the Cu/C-solid and Cu/C-fragment were weak, implying less accumulation of *CO. In comparison, the peak intensity of *CO_atop_ (2058 cm^−1^) on the Cu/C-cavity was stronger than that over Cu/C-solid and Cu/C-fragment. The results suggested that *CO_atop_ intermediates were accumulated on the Cu/C-cavity, indicating that the cavity structure could enrich the local concentration of *CO intermediates.^47^ Overall, the *in situ* Raman results revealed that the cavity structure exhibited a higher coverage of *CO_atop_ compared to the solid and fragment structure, thus accelerating the process of *CO dimerization, resulting in superior CO_2_RR selectivity of C_2+_ products.

To further verify that the local high concentration of *CO could promote the rate of C–C coupling, the Gibbs free energy of the *CO dimerization step^48^ was studied using DFT (Fig. 5d, S29 and S30†). The Gibbs free energy value for the C–C coupling of *CO was found to be 0.74 eV at low *CO coverage, and decreased to 0.38 eV at high *CO coverage. The results suggested that a lower energy barrier for the C–C coupling reaction appeared at high *CO coverage. Thus, the probability of C–C coupling could be promoted by a high *CO coverage, which is consistent with our experimental results.

## Conclusions

In summary, we discussed the correlation between the geometric structures of catalysts and the selectivity of C_2+_ products. We have demonstrated that cavity nanoreactors showed a significantly improved electrochemical CO2RR performance. The Cu/C-cavity exhibited a high C_2+_ FE of 80.5% and C_2+_/C_1_ selectivity ratio of 8.1, which is much higher than that over the Cu/C-solid and Cu/C-fragment. In addition, due to the highly dispersed Cu on the carbon support, the stability of Cu/C-cavity was better than that of most reported pure copper-based nanoreactor catalysts. The results of FEM simulation, *in situ* Raman and DFT supported that the cavity of nanoreactors enriched the local concentration of *CO intermediates, thus promoting the C–C coupling reactions. This research underscored the potential application of functionalized nanoreactors in the highly selective electrosynthesis of valuable fuels from CO_2_, while shedding light on the morphology and composition of catalysts having a significant effect on the performance of the CO_2_RR.

## Data availability

The authors declare that all data supporting the findings of this study are available within the paper [and its ESI†].

## Author contributions

M. W., W. X., H. H. W. and B. X. H. proposed the project, designed the experiments, and wrote the manuscript. M. W. performed the whole experiment. S. Q. J., T. Y., X. D., D. W. Z., and M. H. F. performed the analysis of experimental data. W. X., H. H. W., M. Y. H. and B. X. H. co-supervised the whole project. All authors discussed the results and commented on the manuscript.

## Conflicts of interest

The authors declare no competing interests.

## Supplementary Material

SC-015-D4SC01735H-s001
