# Supplementary material for: Enhancing C2+ product selectivity in CO2 electroreduction by enriching intermediates over carbon-based nanoreactors
Source: Chem Sci. 2024 May 1;15(22):8451–8. doi: 10.1039/d4sc01735h (PMC11151859; doi:10.1039/d4sc01735h)
Supplement: SC-015-D4SC01735H-s001 [file SC-015-D4SC01735H-s001.pdf]

Electronic Supplementary Material (ESI) for Chemical Science.
This journal is © The Royal Society of Chemistry 2024

Supporting Information

Enhancing C_{2+} product selectivity in CO_2 electroreduction by enriching intermediates over carbon-based nanoreactor

Min Wang[a,b], Chunjun Chen[a,b], Shuaiqiang Jia[a,b], Shitao Han[a,b], Xue Dong[a,b], Dawei Zhou[a,b], Ting Yao[a,b], Minghui Fang[a,b], Mingyuan He[a,b], Wei Xia*[a,b], Haihong Wu*[a,b], Buxing Han[a,b,c]*

[a] Shanghai Key Laboratory of Green Chemistry and Chemical Processes, State Key Laboratory of Petroleum Molecular & Process Engineering, School of Chemistry and Molecular Engineering, East China Normal University, Shanghai 200062, China

[b] Institute of Eco-Chongming, 20 Cuiniao Road, Chenjia Town, Chongming District, Shanghai 202162, China

[c] Beijing National Laboratory for Molecular Sciences, CAS Laboratory of Colloid and Interface and Thermodynamics, CAS Research/Education Center for Excellence in Molecular Sciences, Center for Carbon Neutral Chemistry, Institute of Chemistry, Chinese Academy of Sciences, Beijing 100190, China

* Corresponding author. E-mail: wxia@chem.ecnu.edu.cn (W. Xia), hhwu@chem.ecnu.edu.cn (H. Wu), hanbx@iccas.ac.cn (B. Han)

Experimental Section

Materials

1,2,3-triazole and dopamine hydrochloride (DA) were purchased from Aladdin Co., Ltd. (Shanghai, China). Copper nitrate trihydrate ($Cu(NO_3)_2 \cdot 3H_2O$, 99%), N, N-dimethylformamide (DMF) were obtained from Sinopharm Chemical Reagent Co., ltd. Nafion N-117 membrane (0.180 mm thick, ≥ 0.90 meg/g exchange capacity), Nafion D-521 dispersion (5% w/w in water and 1-propanol, ≥ 0.92 meg/g exchange capacity) and Toray carbon paper (CP, TGP-H-60, 19 cm×19 cm), were provided by Alfa Aesar China Co., Ltd. (USA). All chemical reagents and solvents in this work were analytical grade and used without further purification.

Synthesis of MET-5

3 mmol $Cu(NO_3)_2 \cdot 3H_2O$ and 9 mmol 1,2,3-triazole were dissolved in 30 mL DMF. After vigorous stirring for 1 h, the mixture was transferred to Teflon-lined stainless-steel autoclave and kept at 100 °C for 18 h. Finally, the precipitate was thoroughly washed with DMF, and dried at 60 °C for 12 h under vacuum.

Synthesis of MET-5@PDA

300 mg MET-5 was dispersed in 300 mL 10% ethanol-water solution under 10 min sonication to obtain a homogeneous mixture, followed by adding 90 mg DA. The mixture was stirred at room temperature for 6 h to obtain MET-5@PDA. Finally, the precipitate was thoroughly washed with ethanol and DI water, and dried at 60 °C for 12 h under vacuum.

Synthesis of Cu/C-cavity, Cu/C-solid, and Cu/C-fragment

The MET-5 was annealed at 600 °C for 2 h with a heating rate of 5 °C min^{-1} under a 5% H_2/Ar flow to obtain the Cu/C-solid catalyst. The MET-5@PDA was reduced at 600 °C for 2 h with a heating rate of 10 °C min^{-1} under a flowing 5% H_2/Ar atmosphere for 2 h, and the resulting material was named Cu/C-cavity. The MET-5@PDA was reduced at 600 °C for 5 h with a heating rate of 2 °C min^{-1} under a 5% H_2/Ar flow to obtain the Cu/C-fragment catalyst.

Materials Characterization

The morphology of the above samples was characterized by scanning electron microscopy (SEM) S-4800 and transmission electron microscopy TEM (JEM-2100F). Power X-ray diffraction (XRD) patterns were obtained on an X-ray diffractometer (Model D/MAX2500, Rigaka) with Cu-Kα radiation at a scan speed of 10°/min. X-ray photoelectron spectroscopy (XPS) analysis was performed on the Thermo Scientific ESCA Lab 250Xi with a 200 W monochromated Al Kα radiation. The Cu contents were determined by inductively coupled plasma optical emission spectroscopy (ICP-OES) (Optima 8300, Perkin-Elmer). Raman spectra were collected on a confocal Raman microscope (Thermo Scientific DXR2) with a 785 nm solid laser as an excitation source (200 ~ 3000 cm^{-1}). The X-ray absorption spectroscopy (XAS) experiments were carried out at the 4B9A beamline at Shanghai Synchrotron Radiation Facility, China.

Electrochemical experiments

First, 2 mg catalyst was dispersed into 500 uL isopropanol and 25 uL 5% Nafion D-521 under 15 min sonication to obtain homogeneous ink. Then, the ink was spread onto the carbon paper (1×1 cm^{-2}). Electrochemical experiments were conducted on the CHI 660E electrochemical workstation (Shanghai CH Instruments Co., China). The experiments were conducted at room temperature. All the potential (*vs*. Ag/AgCl) in this work were converted to RHE using the following equation:

$$E_{RHE} = E_{Ag/AgCl} + 0.197 + 0.059 \times pH$$

Electrocatalytic CO_2RR measurements: Electrocatalytic CO_2 reduction experiments were conducted in a typical H-type cell with a three-electrode system (the working electrode, the platinum gauze counter electrode, and the Ag/AgCl reference electrode). The Nafion-117 exchange membrane was used to separate the cathode and anode compartments. 30 ml CsI solution (0.1 M) and K_2SO_4 solution (0.1 M) were used as the cathode and anodic electrolytes, respectively. Before electrolysis, the cathodic electrolyte was saturated with CO_2. During the electrolysis process, the CO_2 flow was kept constant at 15 mL·min^{-1}, and slight magnetic stirring was performed to obtain uniform electrolyte. Linear sweep voltammetry (LSV) measurements: LSV scans were conducted in an H-type cell. 0.1 M CsI solution was used as the cathode and 0.1 M K_2SO_4 solution was used as anodic electrolytes. At the start of each experiment, the electrolyte was bubbled with N_2 or CO_2 at least 30 min. LSV measurements were conducted at a range of applied potentials between 0 and -1.4 V *vs.* RHE. (scan rate 20 mV s^{-1}).

Electrochemical active surface area measurement (ECSA): Electrochemical active surface area measurements for the catalytic electrodes were determined by double-layer capacitances (C_{dl}). Due to the value of C_{dl} is a direct ratio to the electrochemically active surface area, C_{dl} could be determined by measuring the capacitive current associated with double-layer charging from the scan-rate dependence of cyclic voltammogram (CV). In the non-Faradaic region, the CV ranged from 0.5 V to 0.6 V *vs*. RHE. The scan rates were set to 40, 60, 80, 100, and 120 mV s^{-1}. The value of C_{dl} was estimated by plotting of the Δj (j_a-j_c) at 0.55 V (*vs*. RHE) vs the scan rates, where the j_a and j_c were the anodic and cathodic current density, respectively. The slope is the C_{dl} value.

Electrochemical impedance spectroscopic (EIS) measurements: The EIS measurements were conducted at the open circuit potential in 0.1M K_2SO_4 solution (CO_2 saturated) with an amplitude of 5 mV of 0.01 to 10^6 Hz.

Product analysis: Gas bag was used to collect gaseous products, and the gaseous products were quantified by gas chromatography (GC, Agilent-8890) equipped with FID and TCD detector (argon as the carrier gas). The Faradaic efficiencies (FE) of gaseous products was calculated by the following equation:

$$FE = \frac{n \times F \times n_{product}}{Q} \times 100\%$$

Where Q is total electric quantity, F is the Faraday constant (96485 C mol^{-1}), n is the transfer electron number in the reaction, $n_{product}$ is the moles of product.

The liquid products in D_2O (DMSO and phenol as aninternal standard) were analyzed by a nuclear magnetic resonance (NMR) spectrometerr (Bruker; Ascend 400 MHz).

Finite element method simulations

In the experimental study of electrocatalytic CO_2 reduction, there are complex interactions between CO_2 feedstock, C_1 intermediate and products, C_{2+} intermediate and products, and also adsorption and desorption interactions, coupling reactions. In this study, the “Chemistry” module, the “Transport of Diluted Species” module, the “Surface Reactions” module, and “Electric Current” module were used to simulate the electrocatalytic CO_2 reduction process.

First, the “Chemistry” module was used to define the CO_2RR intermediate steps. Three chemical species are defined: CO_2, $C_{1,}$ and C_{2+}, each in a bulk and a surface adsorbed form, representing CO_2 feedstock, C_1 intermediate, and C_{2+} product, respectively. Five reactions were defined: three surface adsorption-desorption equilibrium reactions for the three chemical species, as well as two irreversible reactions for the CO_2 reduction into C_1, the C_1–C_1 coupling into C_{2+}. Second, the “Transport of Diluted Species” module was used to solve the

mass transport of the three species. The diffusion constants of CO_2, C_1 (CO assumed), and C_{2+} (C_2H_4 assumed) were taken to be 1.80 × 10^{-9} m^2 s^{-1}[1], 1.00 × 10^{-9} m^2 s^{-1}[2], 1.085 × 10^{-9} m^2 s^{-1}[3]. Finally, the "Surface Reactions" module defines the catalyst surface where the surface reactions take place. The "Electric Current" module in Comsol was used in the electric potential simulation. Cathode at -1.2 V *vs.* RHE (the potential with the best experimental C_{2+}/C_1 selectivity) was connected to the outer surface of the Cu/C-cavity nanoreactor, and an anode at 1.23 V *vs.* RHE was placed 2000 nm away from the Cu/C-cavity nanoreactor. The conductivity of the 0.1 M CsI electrolyte is 14.61 mS/cm.

Computational details

All the calculations are performed in the framework of the density functional theory with the projector augmented plane-wave method, as implemented in the Vienna ab initio simulation package [4]. The generalzied gradient approximation proposed by Perdew, Burke, and Ernzerhof is selected for the exchange-correlation potential [5]. The cut-off energy for plane wave is set to 500 eV. The energy criterion is set to 10^{-5} eV in iterative solution of the Kohn-Sham equation. The Brillouin zone integration is performed using a 5x5x1 k-mesh. All the structures are relaxed until the residual forces on the atoms have declined to less than 0.01 eV/Å.

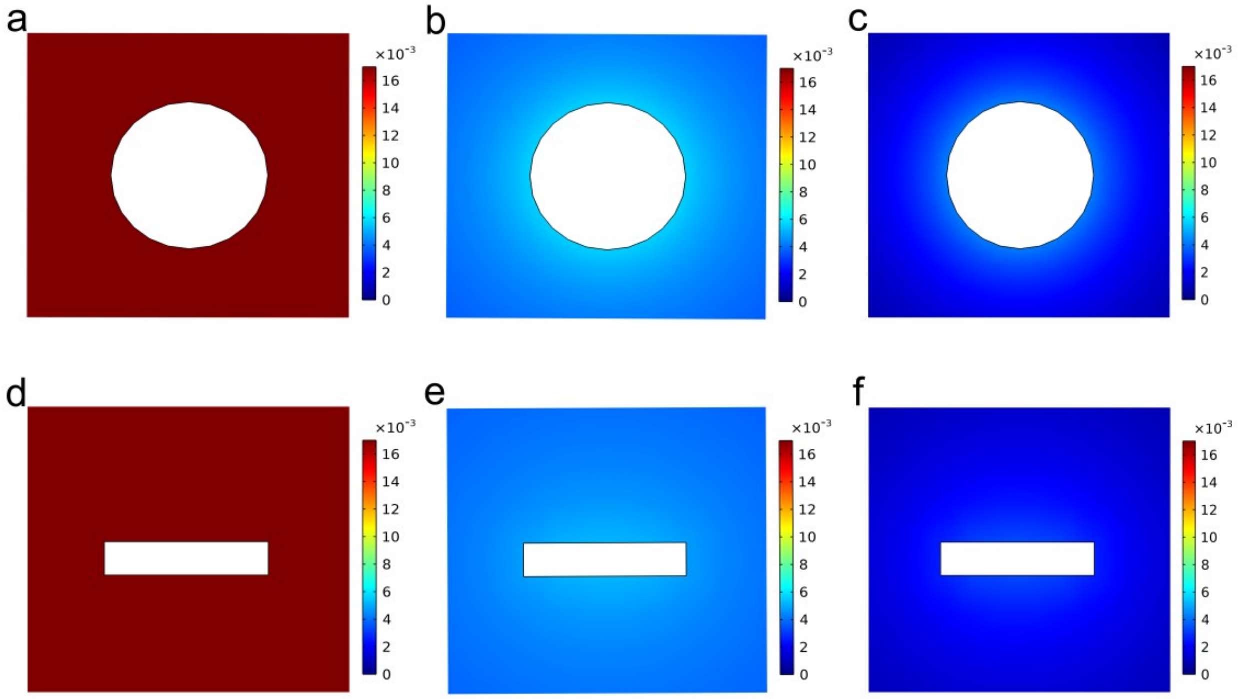

Fig. S1. Computed concentration and flux distribution of species. CO_2 (a), *CO (b) and C_{2+} (c) concentrations (colour scale, in mol/L) and flux distributions (arrows) on the solid model (fully closed structures). CO_2 (d), C_1 (e) and C_{2+} (f) concentrations (colour scale, in mol/L) and flux distributions (arrows) on the fragment model (fully open structures).

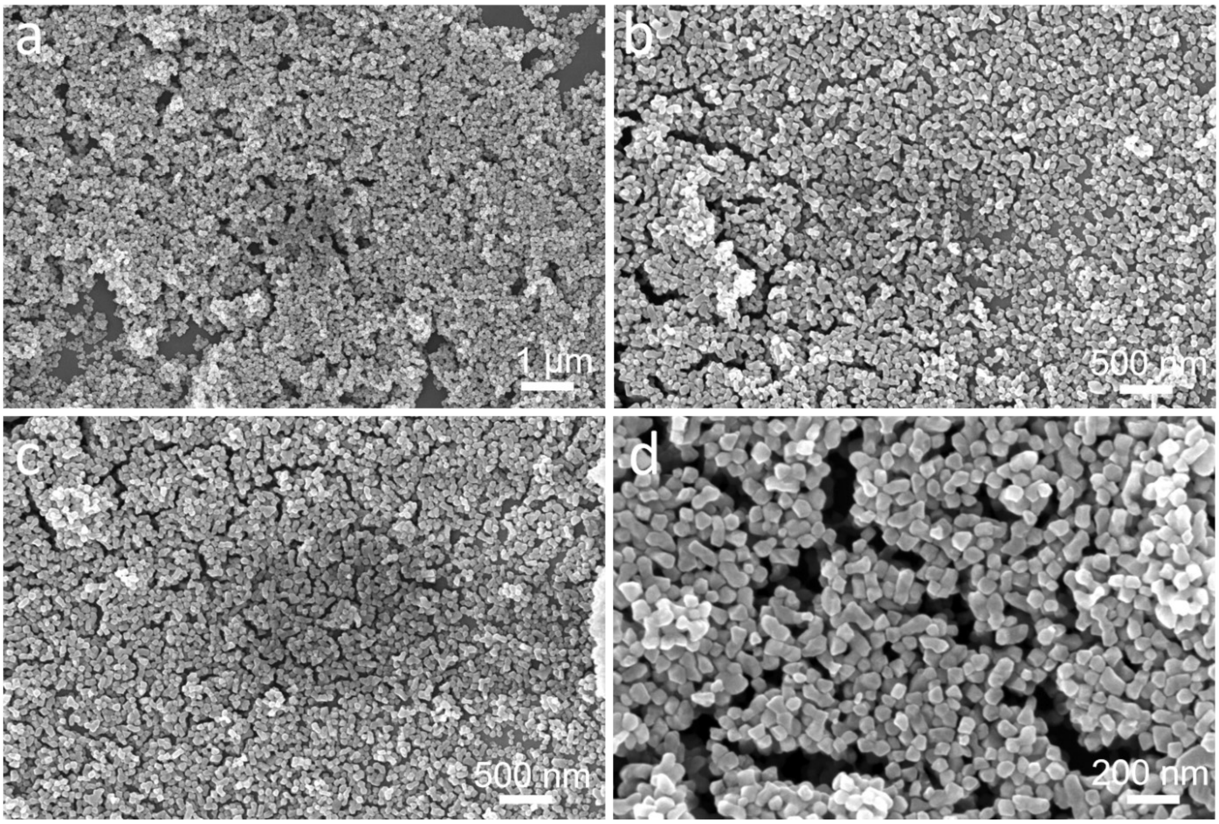

Fig. S2. SEM images of MET-5.

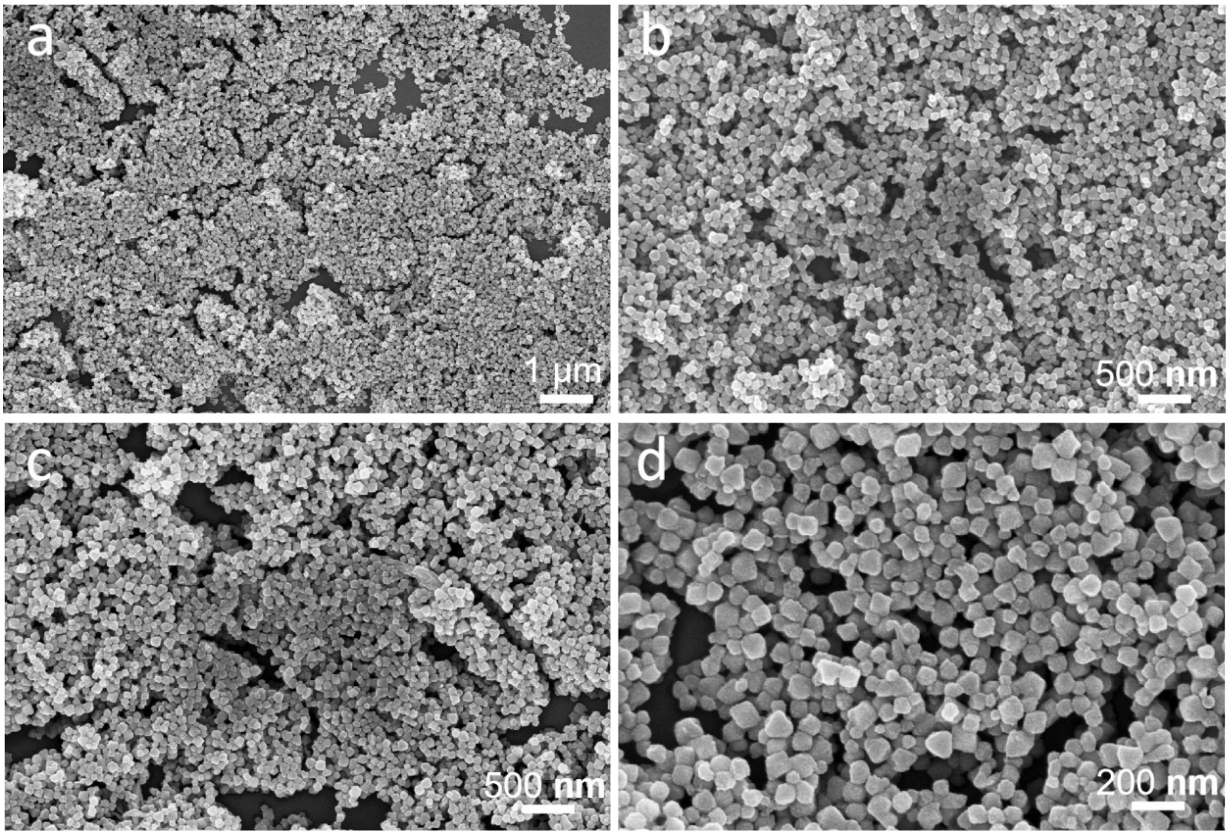

Fig. S3. SEM images of MET-5@PDA.

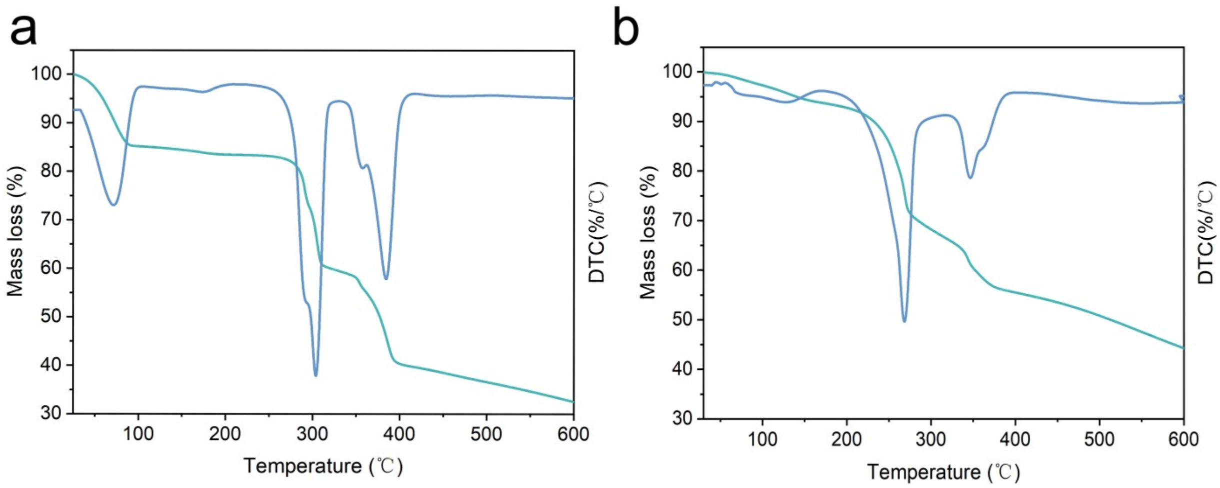

Fig. S4. Thermogravimetric analysis of (a) MET-5 and (b) MET-5@PDA measured from 25 to 600℃ (10℃ min^{-1}) under a nitrogen atmosphere.

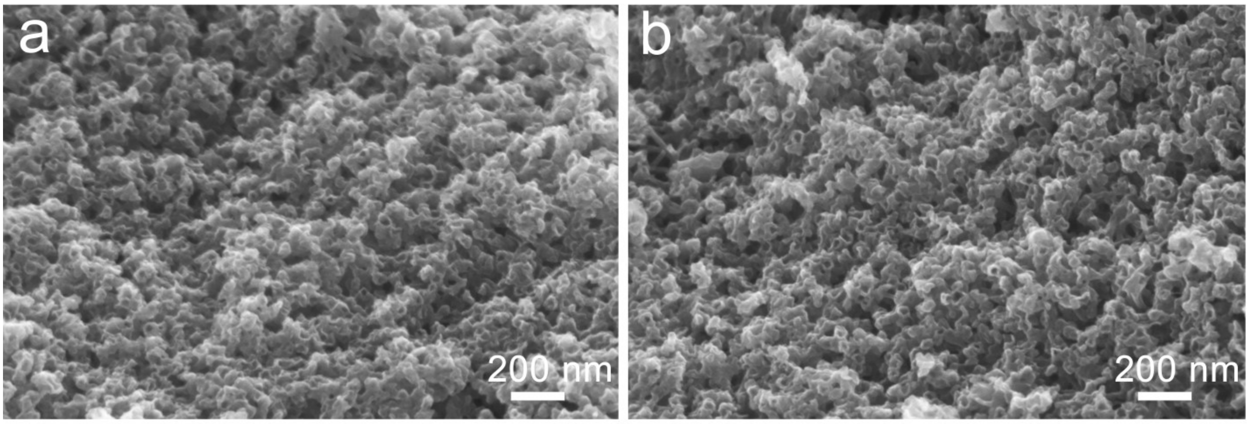

Fig. S5. SEM images of Cu/C-cavity catalyst.

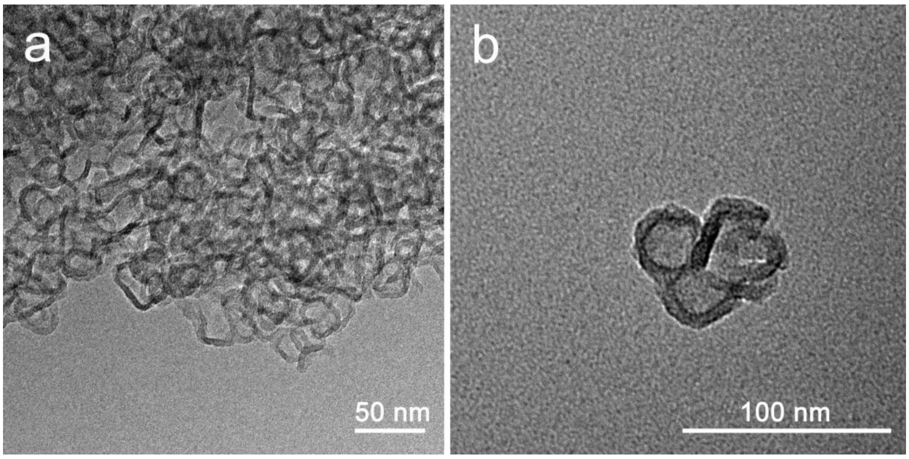

Fig. S6. TEM images of Cu/C-cavity catalyst.

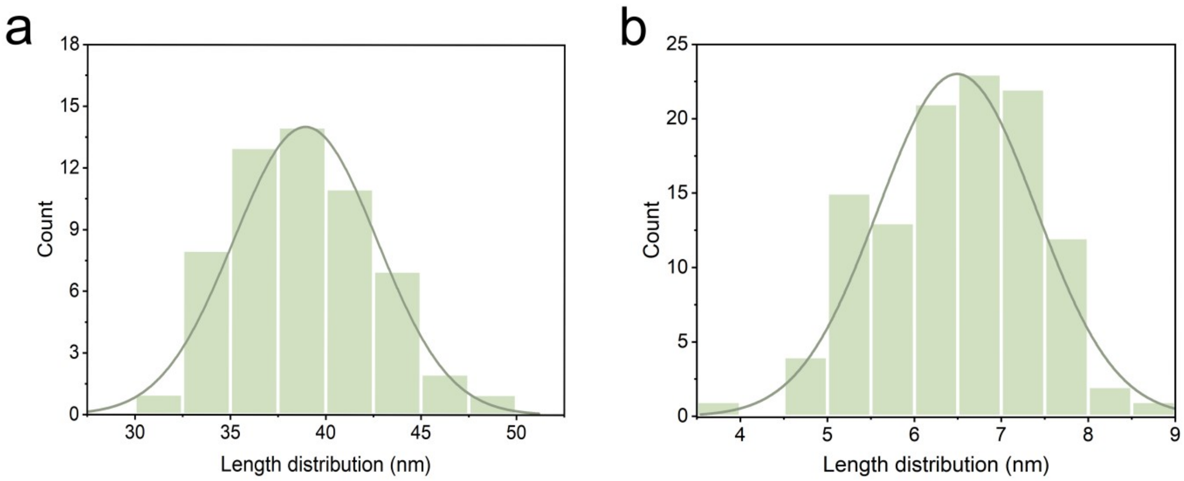

Fig. S7. The particle size and shell thickness distribution of Cu/C-cavity catalyst. It can be seen that the particle sizes and shell thickness of Cu/C-cavity mainly distribute at 35-45 nm and 6-7.5nm, respectively.

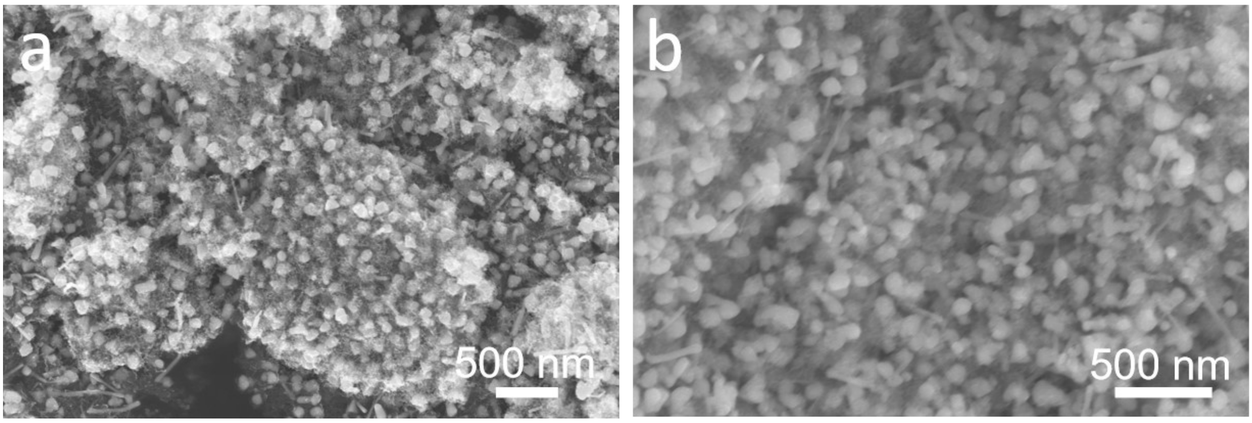

Fig. S8. SEM images of Cu/C-solid catalyst.

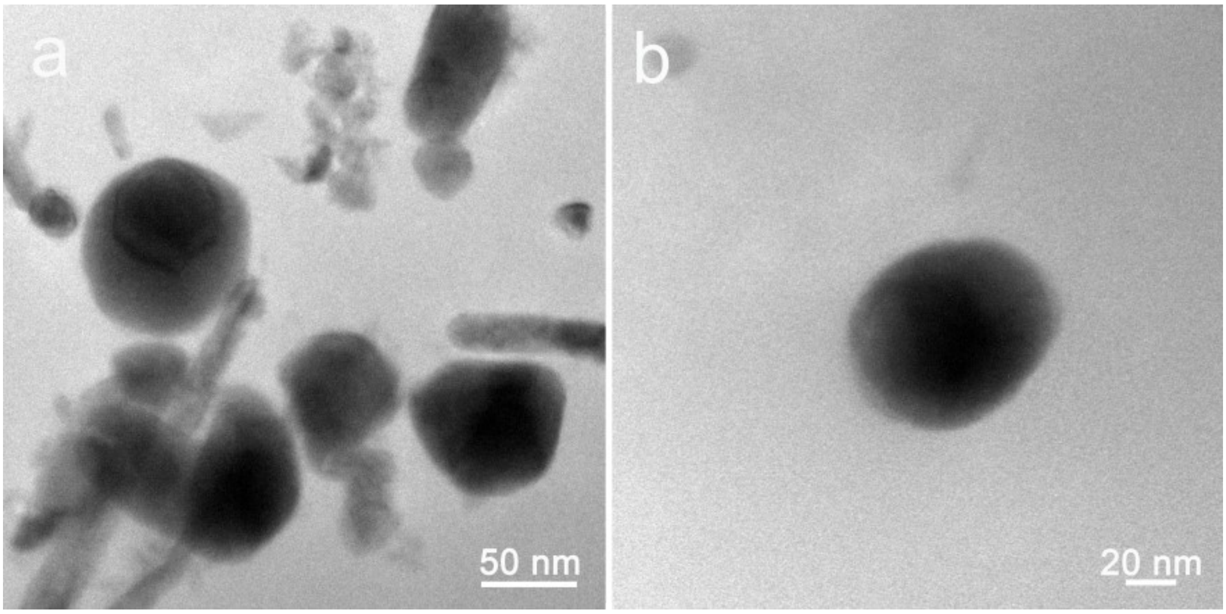

Fig. S9. TEM images of Cu/C-solid catalyst.

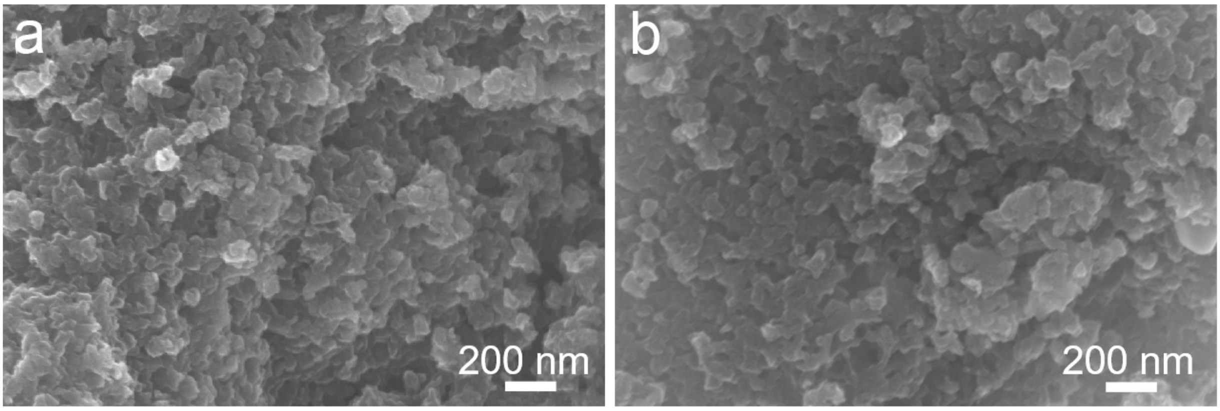

Fig. S10. SEM images of Cu/C-fragment catalyst.

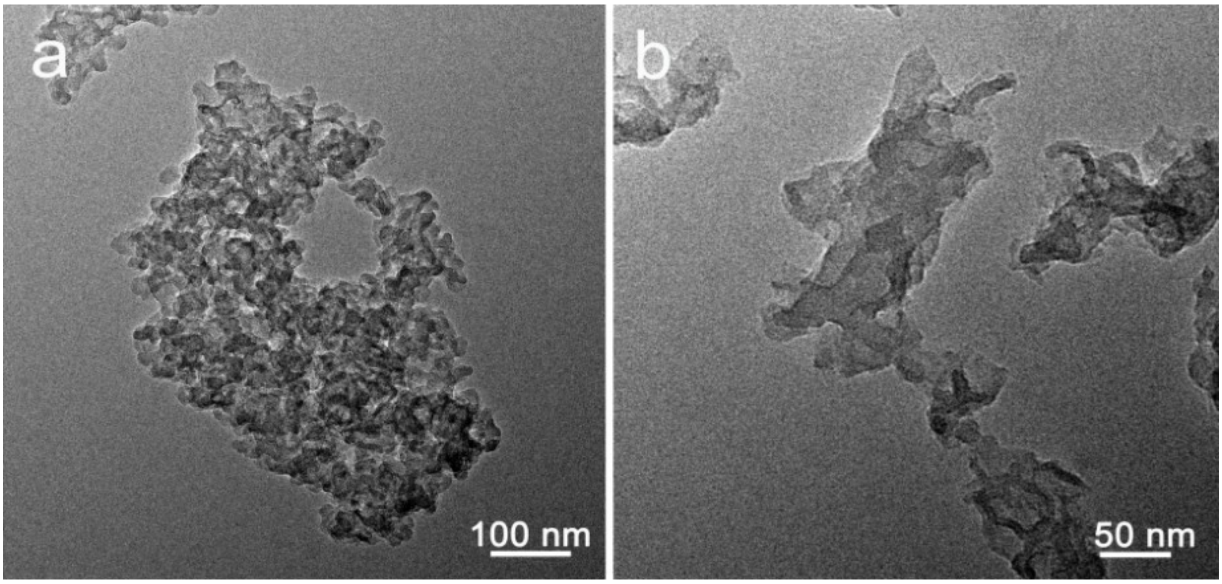

Fig. S11. TEM images of Cu/C-fragment catalyst.

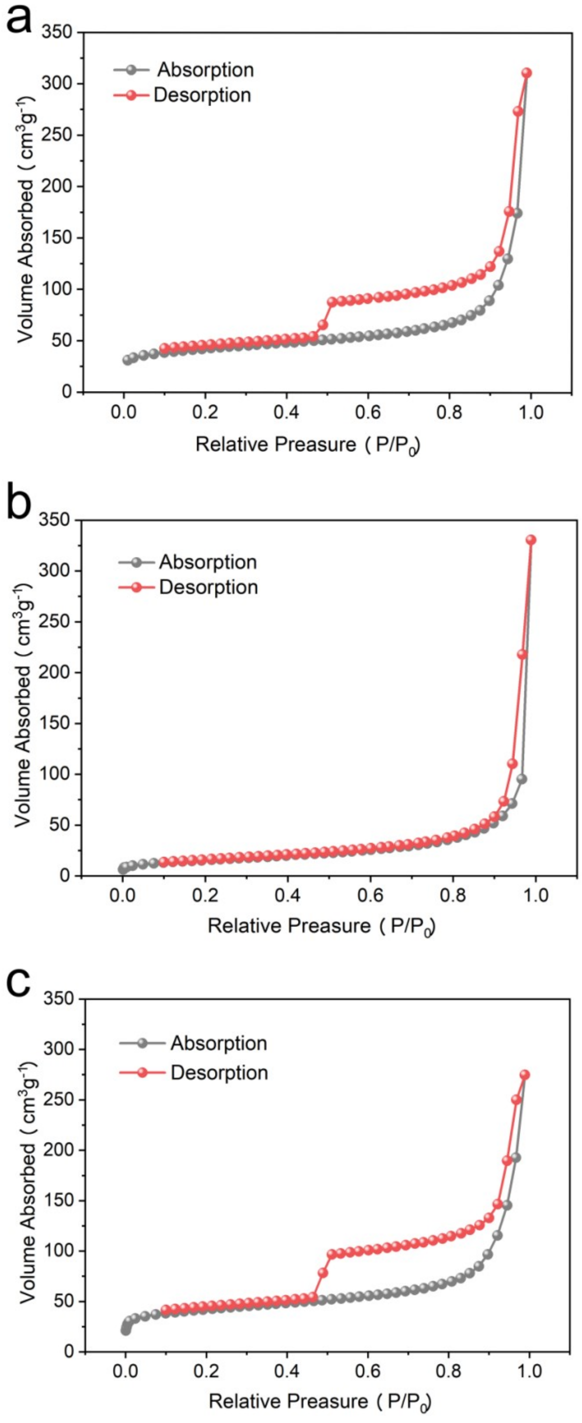

Fig. S12. BET surface area analysis. Nitrogen adsorption-desorption isotherms of (a) Cu/C-cavity, (b) Cu/C-solid and (c) Cu/C-fragment catalysts. The BET surface areas of Cu/C-cavity, Cu/C-solid, and Cu/C-fragment catalysts determined were 144.78m²/g, 56.35m²/g, and 149.21m²/g, respectively.

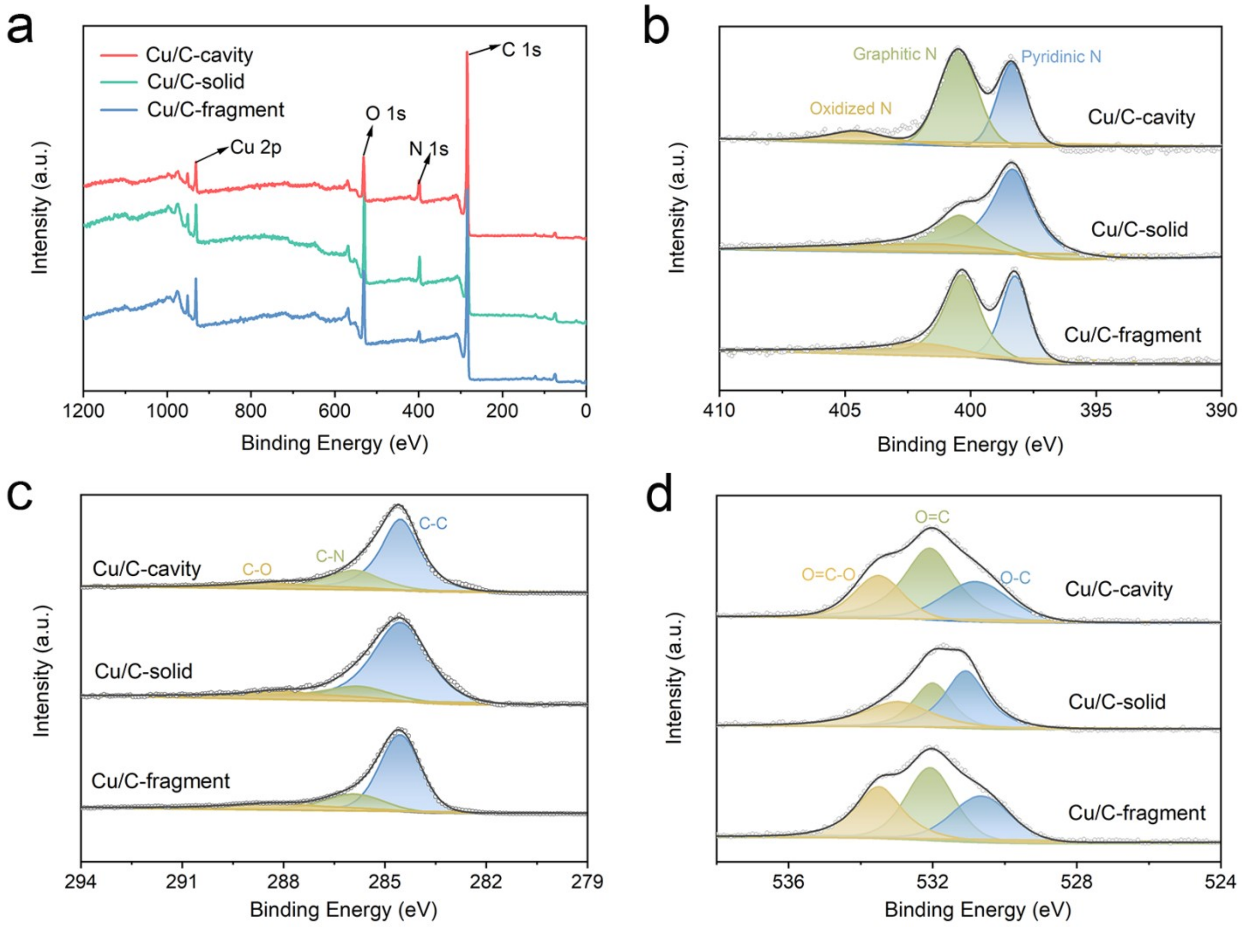

Fig. S13. XPS survey spectra (a), N 1s (b), C 1s (c), and O (d) regions of Cu/C-cavity, Cu/C-solid, and Cu/C-fragment catalysts.

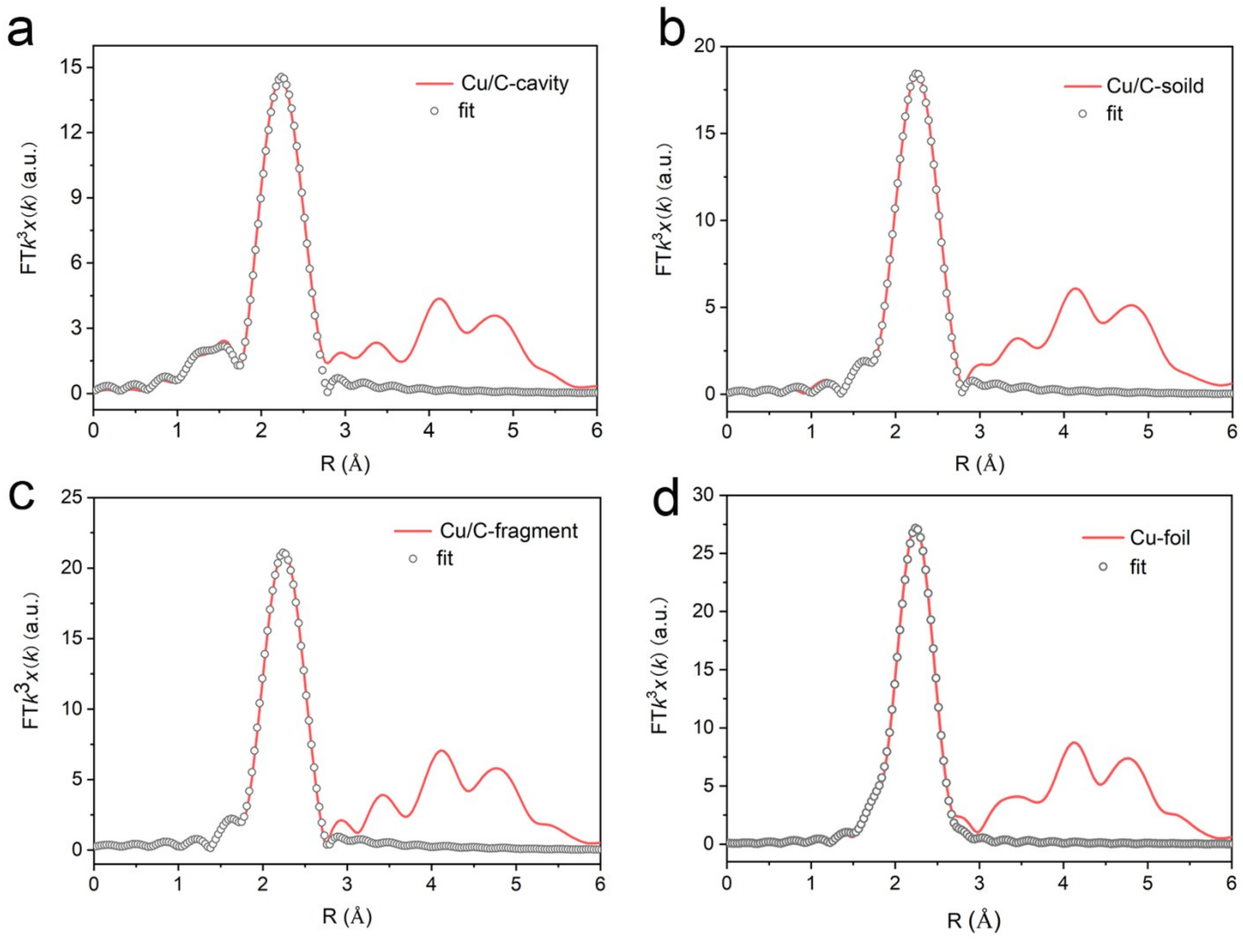

Fig. S14. The corresponding EXAFS fitting curves of (a) Cu/C-cavity, (b) Cu/C-solid, (c) Cu/C-fragment, and (d) Cu foil.

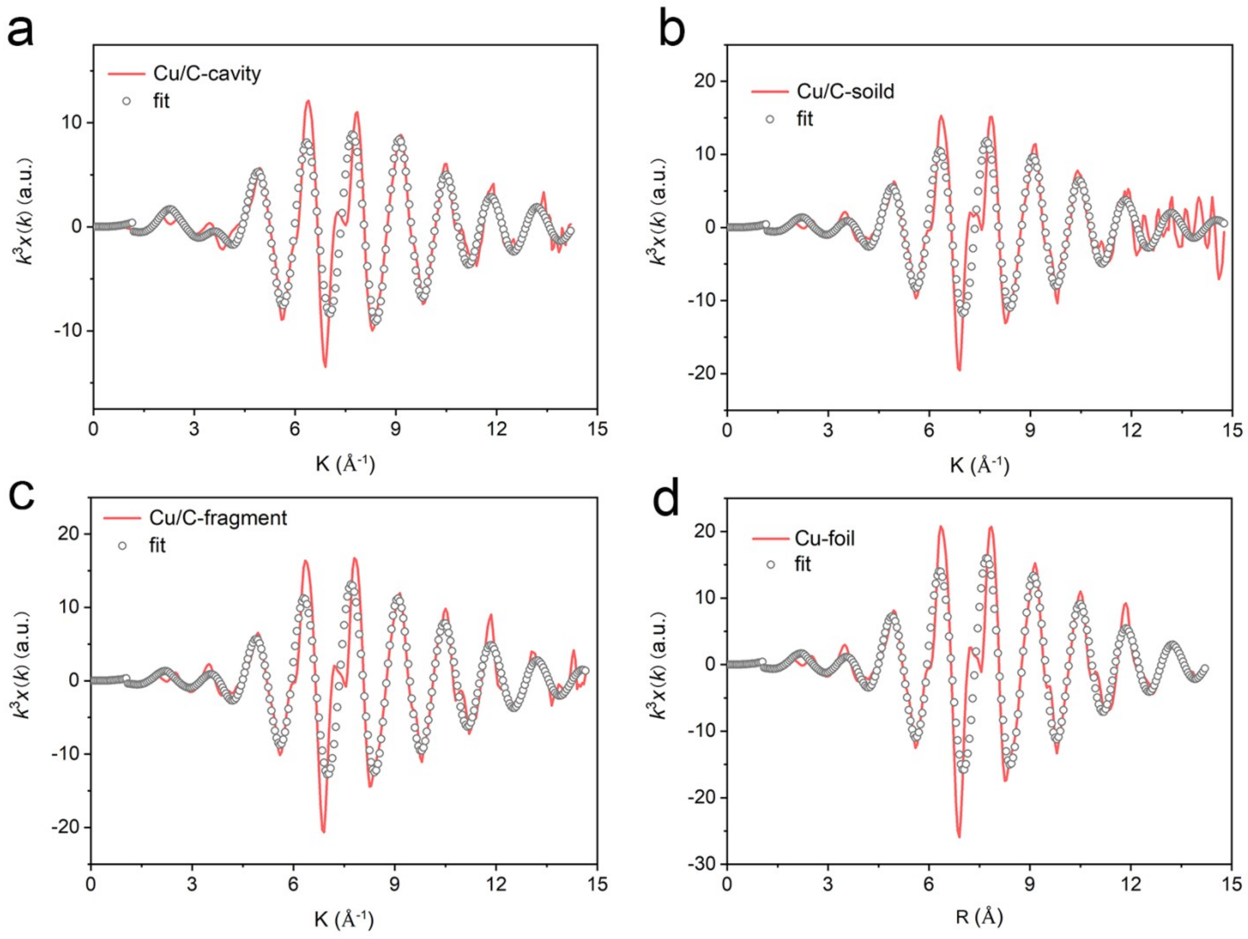

Fig. S15. Cu K edge EXAFS oscillation function in q space of (a) Cu/C-cavity, (b) Cu/C-solid, (c) Cu/C-fragment, and (d) Cu foil.

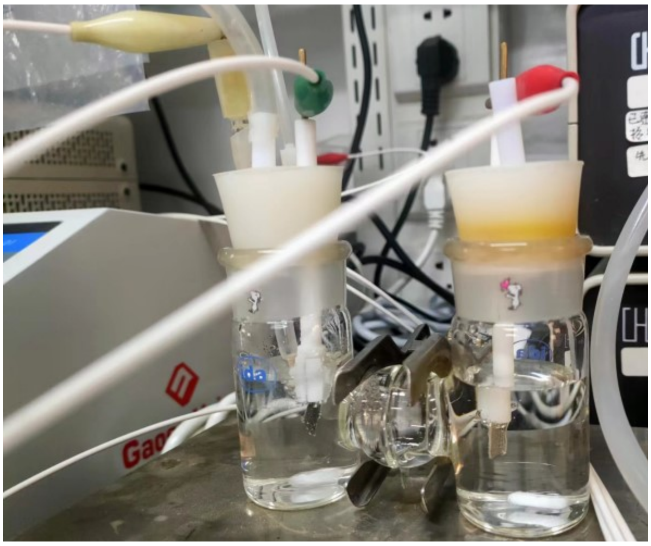

Fig. S16. Photo of the used H-cell.

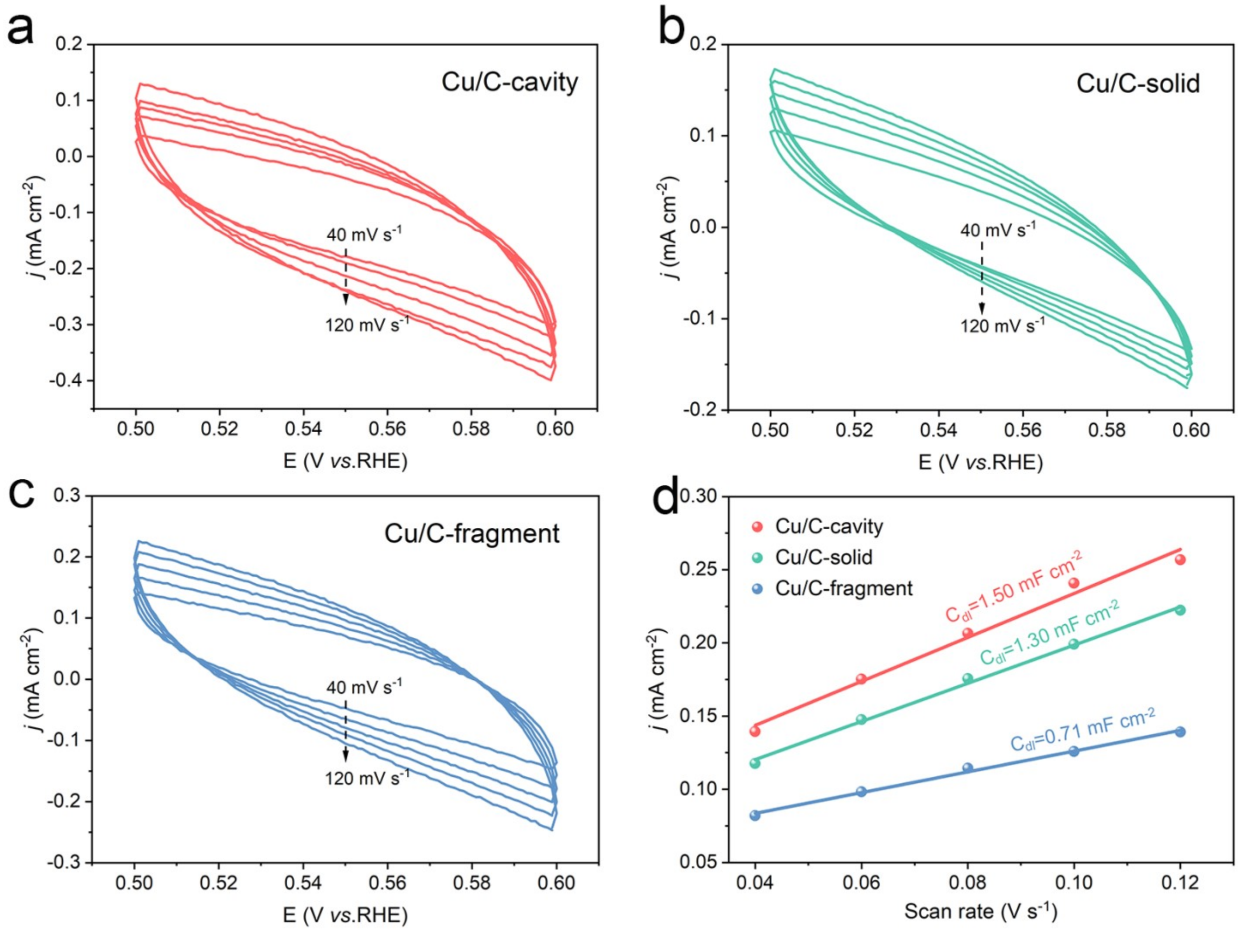

Fig. S17. CV curves with various scan rates and double-layer capacitance of (a) Cu/C-cavity, (b) Cu/C-solid and (c) Cu/C-fragment catalysts.

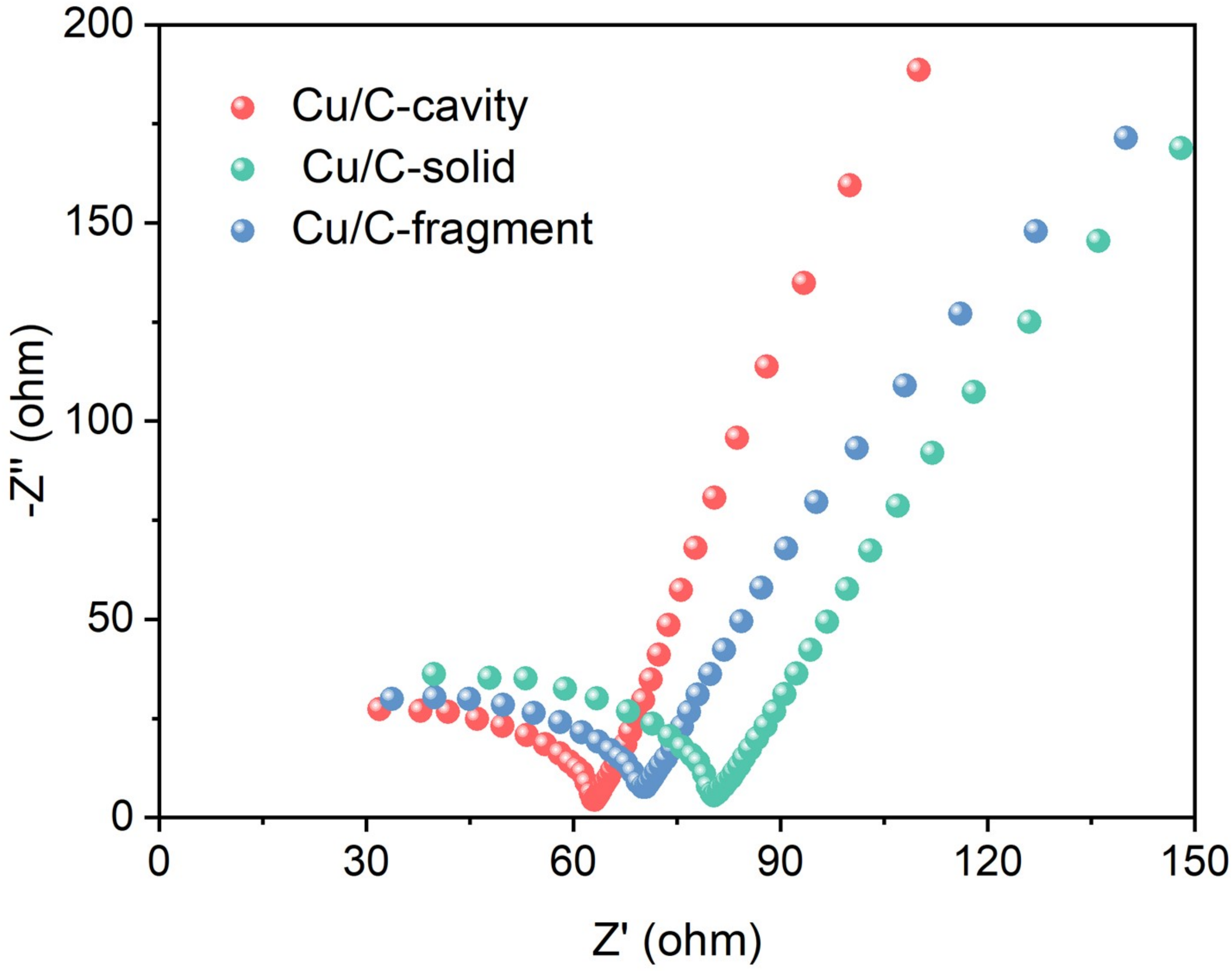

Fig. S18. Nyquist plots of Cu/C-cavity, Cu/C-solid and Cu/C-fragment.

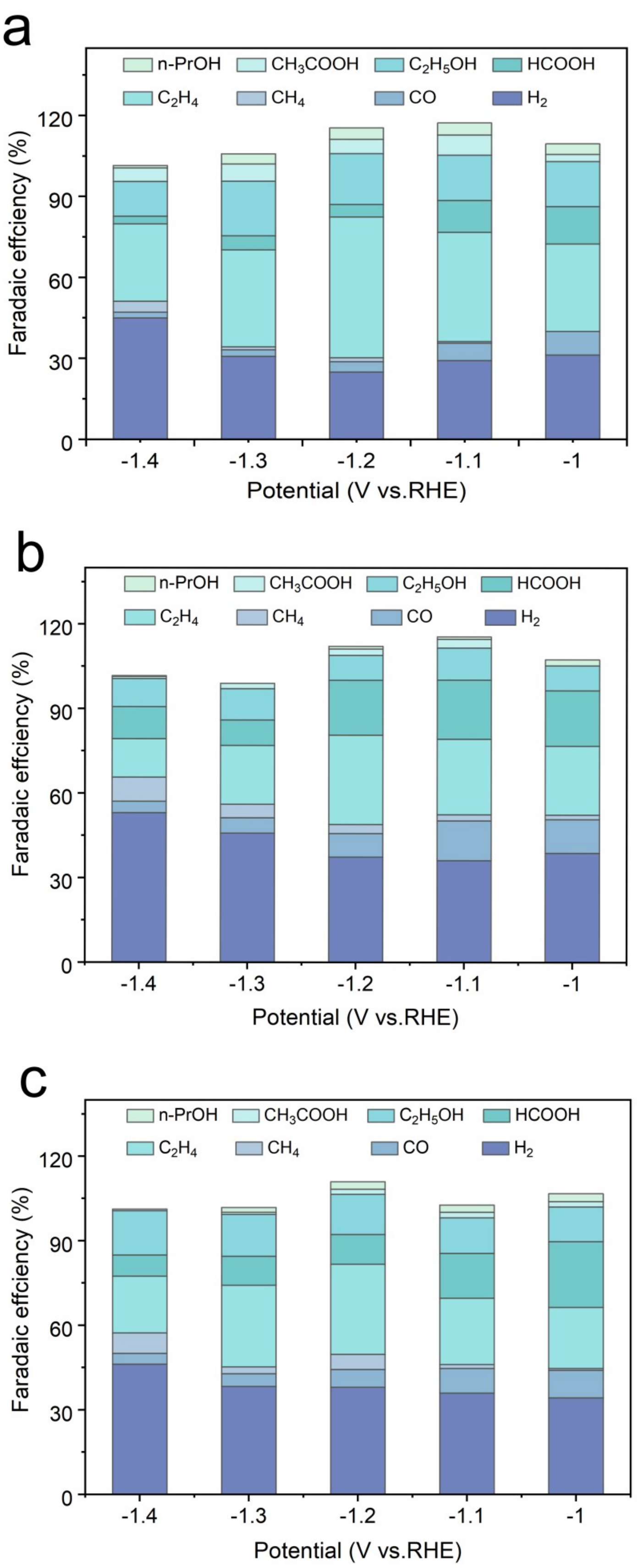

Fig. S19. Faradaic efficiencies of major products in CO_2RR on (a) Cu/C-cavity, (b) Cu/C-solid and (c) Cu/C-fragment catalysts at different applied potentials in 0.1 M CsI in H-cell.

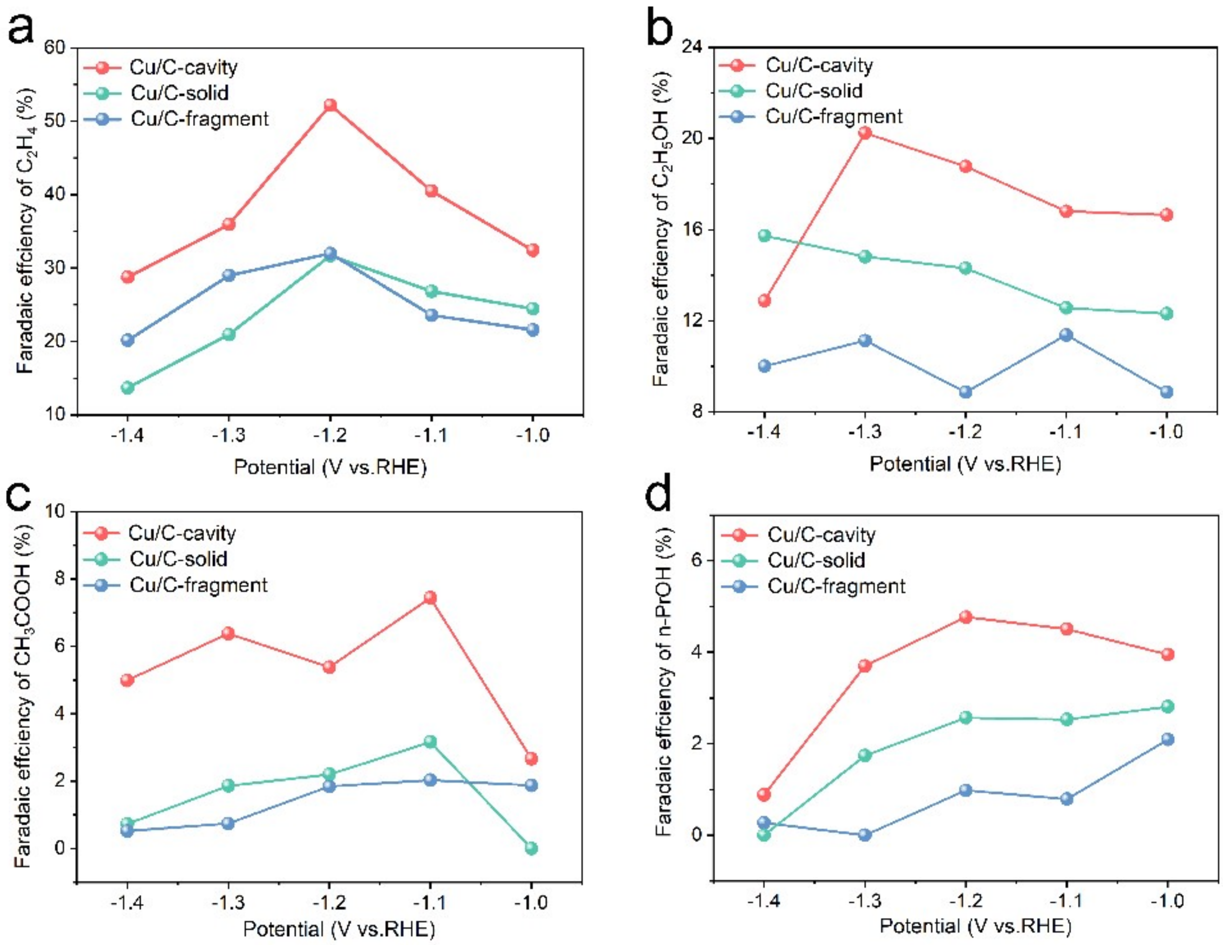

Fig. S20. Faradaic efficiencies of (a) C_2H_4, (b) C_2H_5OH, (c) CH_3COOH, (d) n-PrOH products on the Cu/C-cavity, Cu/C-solid, and Cu/C-fragment in H-cell in CO_2 saturated 0.1 M CsI aqueous electrolyte.

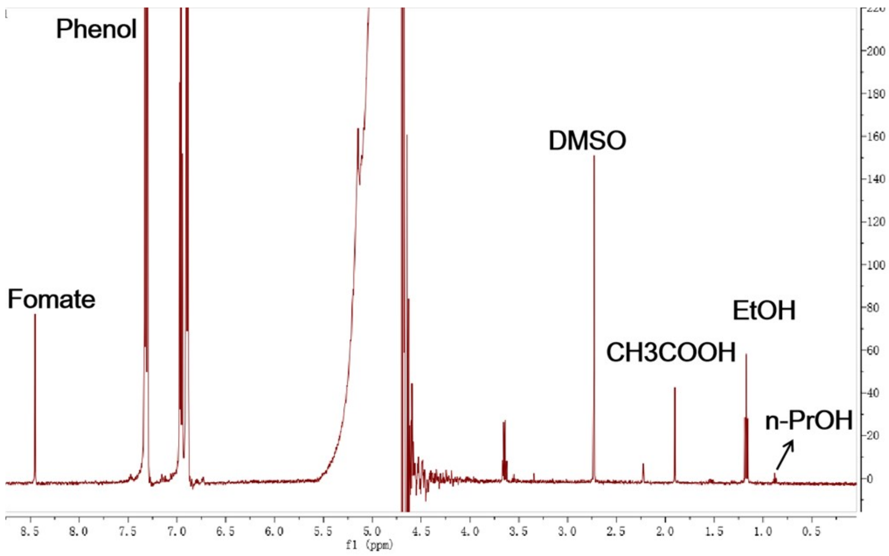

Fig. S21. ^{1}H-NMR spectra of the electrolyte collected at -1.2 V *vs*. RHE for the Cu/C-cavity catalyst in H-cell in CO_2 saturated 0.1 M CsI aqueous electrolyte.

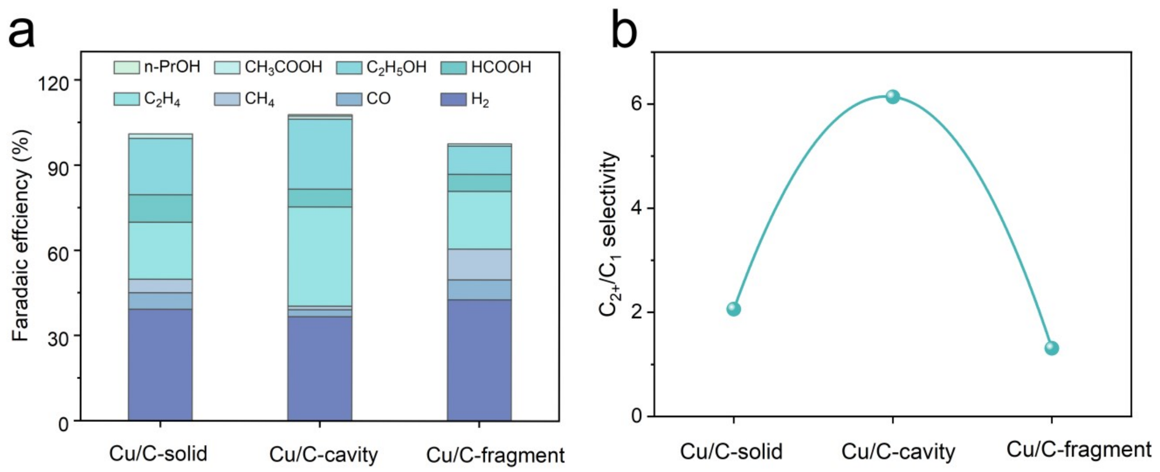

Fig. S22. (a) Faradaic efficiencies of products in CO_2RR on Cu/C-cavity, Cu/C-solid and Cu/C-fragment catalysts at -1.2 V *vs*. RHE in CO_2 saturated 0.1 M $KHCO_3$ aqueous electrolyte in H-cell. (b) C_{2+}/C_1 products selectivity ratio at -1.2 V *vs*. RHE on Cu/C-cavity, Cu/C-solid and Cu/C-fragment catalysts in CO_2 saturated 0.1 M $KHCO_3$ aqueous electrolyte.

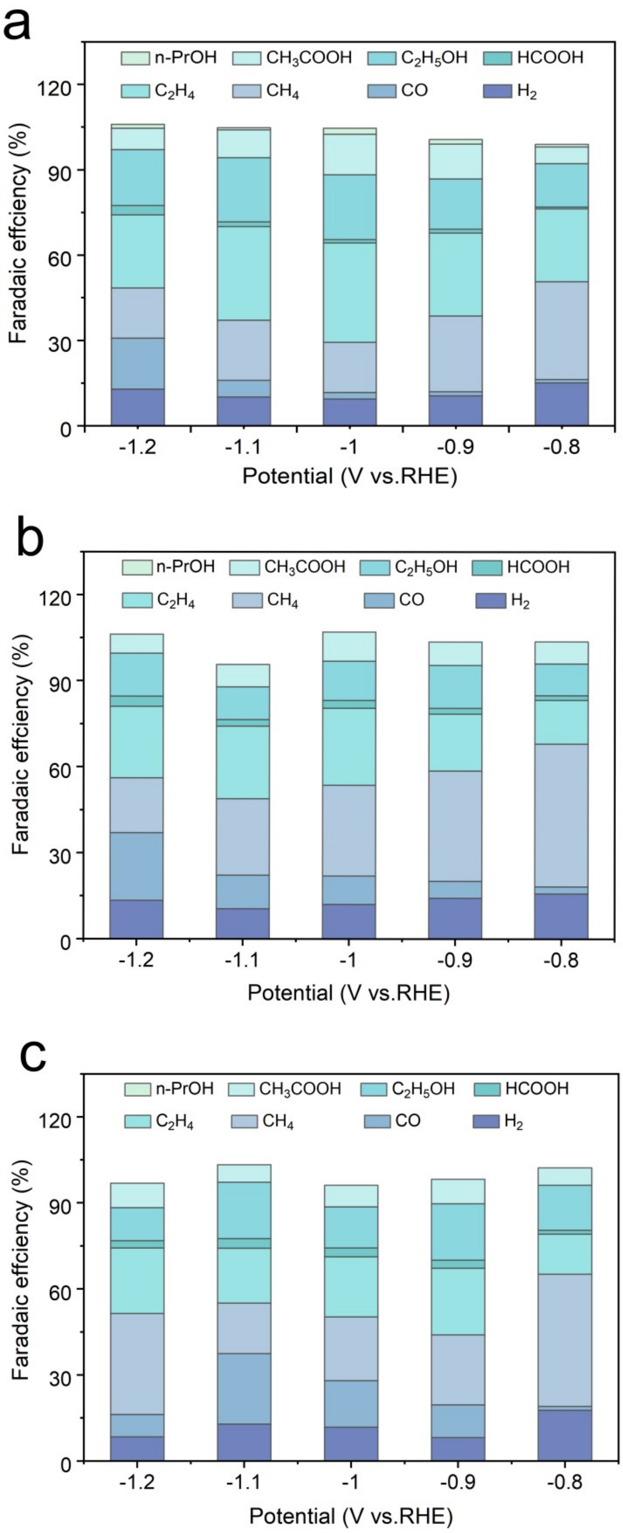

Fig. S23. Faradaic efficiencies of major products in CO_2RR on (a) Cu/C-cavity, (b) Cu/C-solid and (c) Cu/C-fragment catalysts at different applied potentials in 1 M KOH in flow cell.

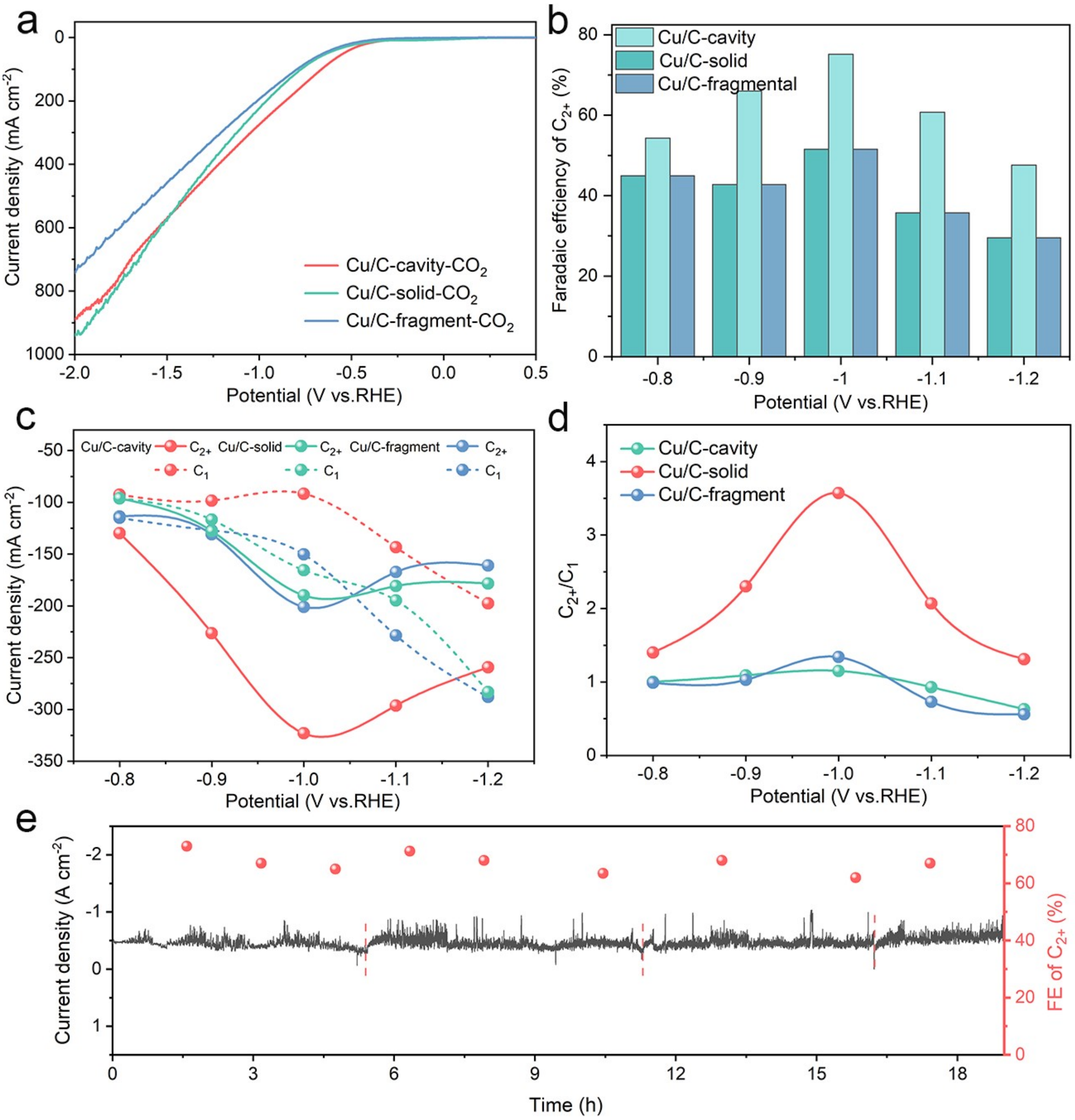

Fig. S24. (a) LSV curves on Cu/C-cavity, Cu/C-solid and Cu/C-fragment in CO_2-saturated electrolytes, (b) C_{2+} FE, (c) C_{2+} and C_1 partial current densities, (d) C_{2+}/C_1 products selectivity ratio from -0.8 V to -1.2 V vs. RHE. The experiments were conducted in 1 M KOH in flow cell, (e) Stability test of Cu/C-cavity at -1.0 V vs. RHE, the dotted line indicate the renewal of the electrolyte.

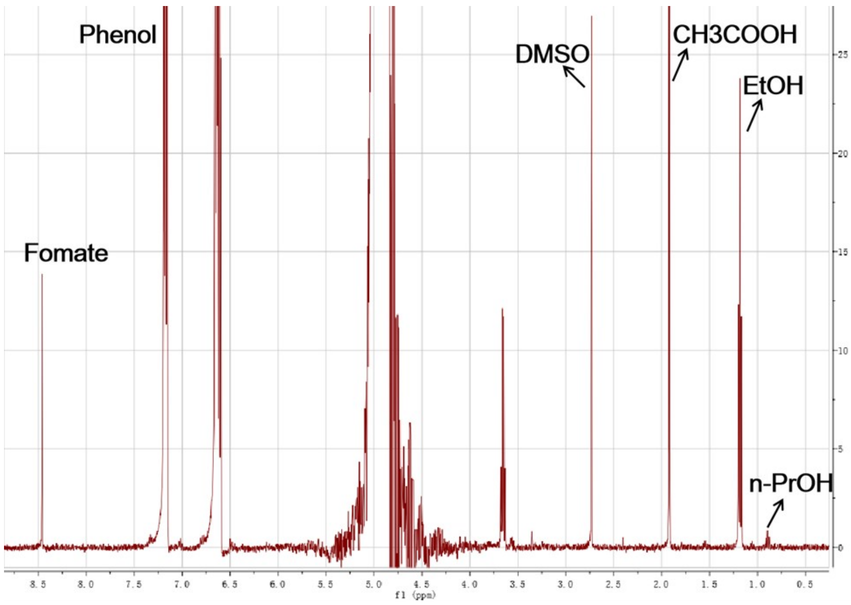

Fig. S25. ^{1}H-NMR spectra of the electrolyte collected at -1.0 V *vs*. RHE for Cu/C-cavity in flow cell.

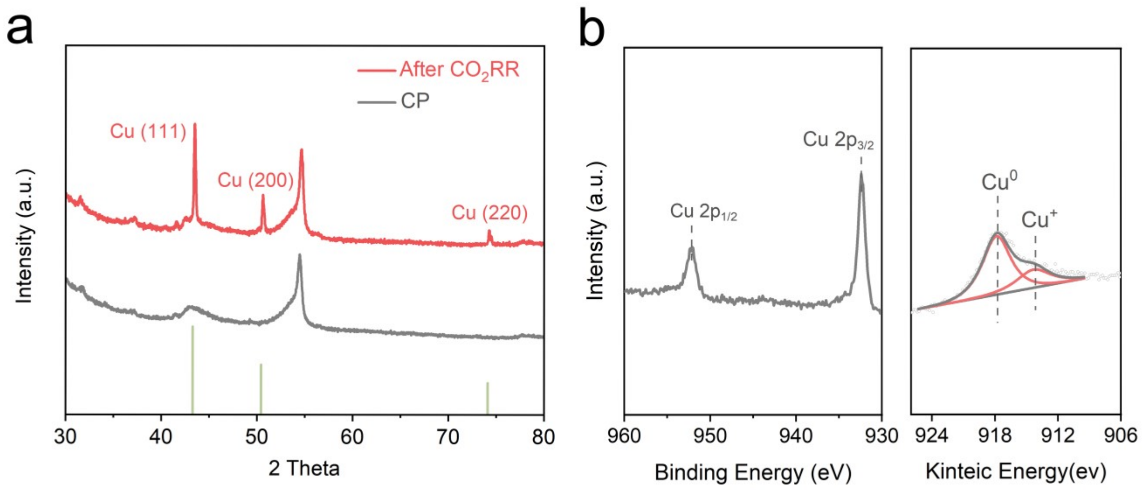

Fig. S26. (a) XRD, (b) Cu-2p and Cu-LMM of the Cu/C-cavity after CO_2RR.

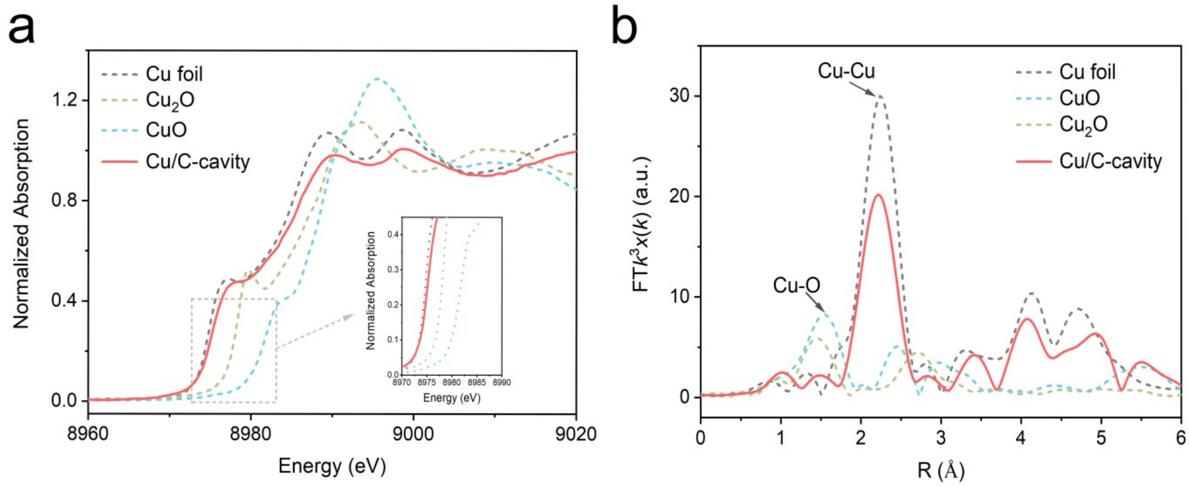

Fig. S27. (a) Cu K-edge XANES, (b) Fourier transformed Cu K-edge EXAFS spectra of the Cu/C-cavity after CO_2RR.

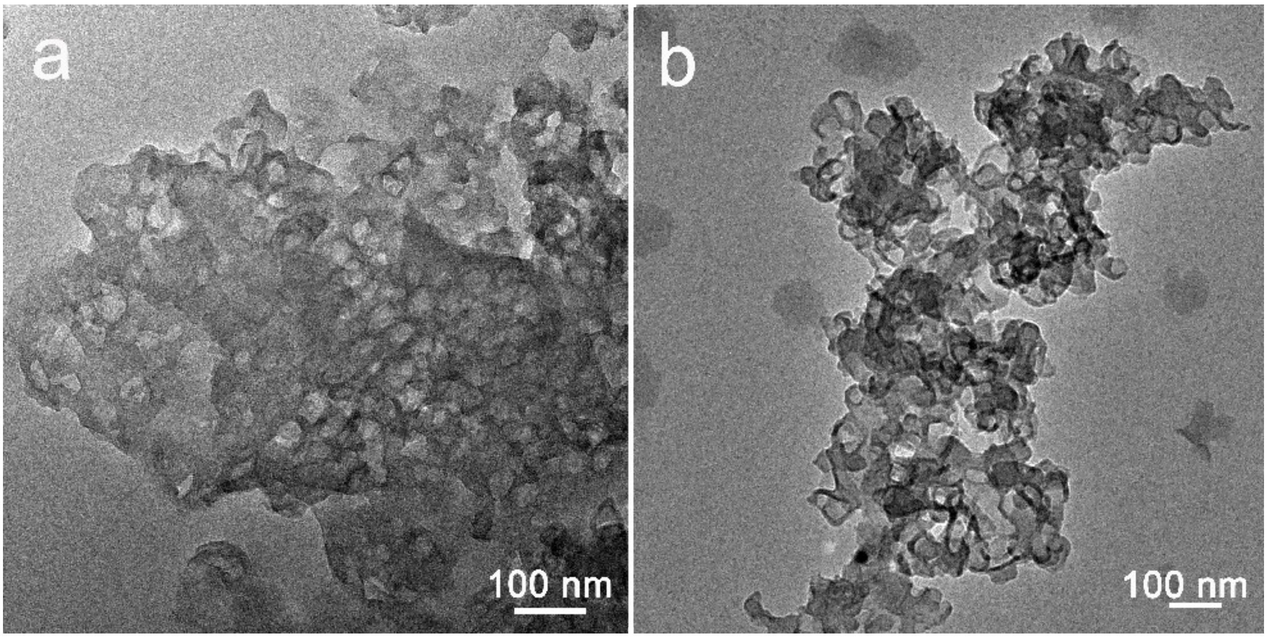

Fig. S28. TEM images of the Cu/C-cavity catalyst after CO_2RR.

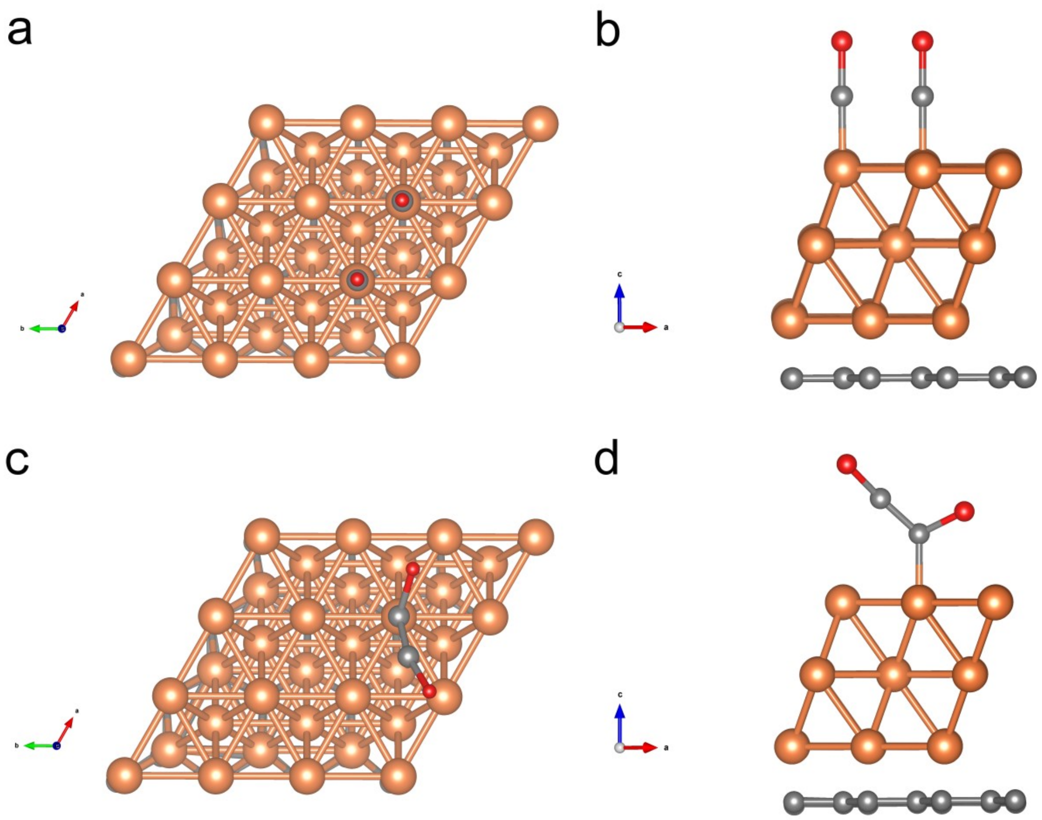

Fig. S29. Adsorption configuration of intermediates on Cu (111) surface at low CO* coverage. Top views of absorption configurations for (a) CO*, (c) OCCO*. Side views of absorption configurations for (b) CO*, (d) OCCO*. The orange, red, grey spheres represent copper, oxygen, carbon, respectively.

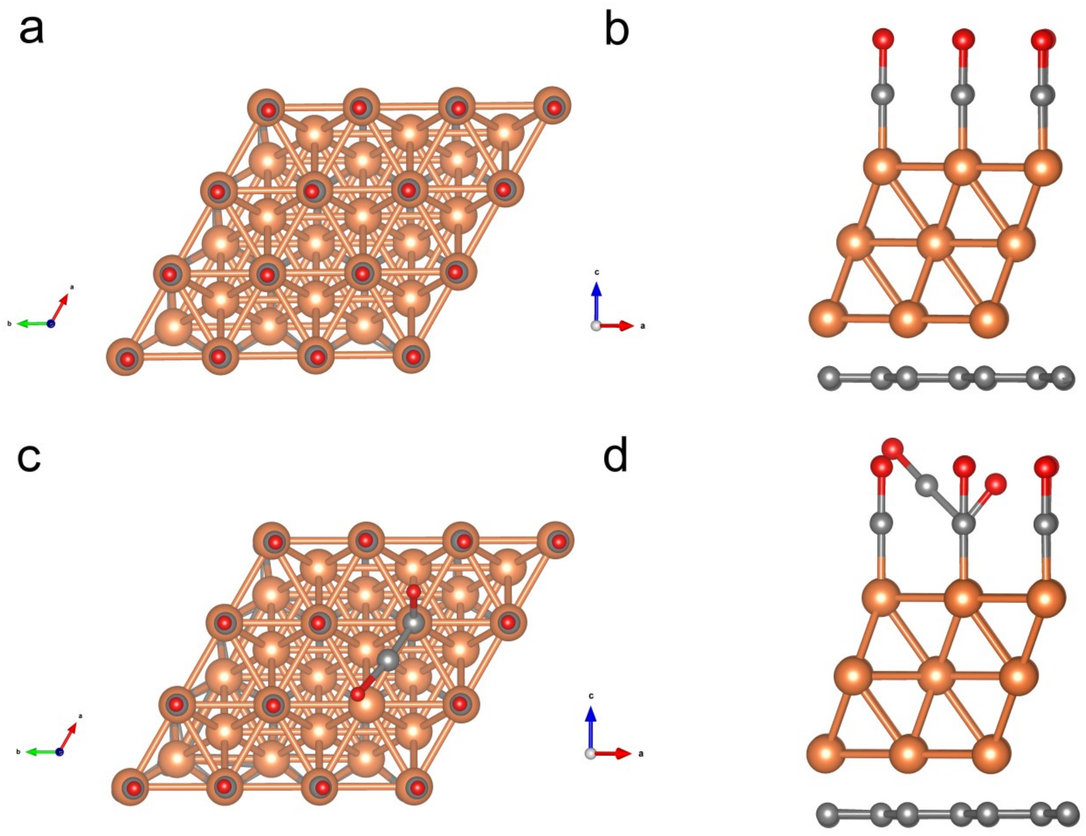

Fig. S30. Adsorption configuration of intermediates on Cu (111) surface at high CO* coverage. Top views of absorption configurations for (a) CO*, (c) OCCO*. Side views of absorption configurations for (b) CO*, (d) OCCO*. The orange, red, grey spheres represent copper, oxygen, carbon, respectively.

Table S1. The C_{2+} partial current density(j_{C2+}) normalised to ECSA of the catalysts.

**Electrocatalyst**	**j_{C2+} (mA cm^{-2})**	**C_{dl} (mF cm^{-2})**	**j_{C2+} normalised to C_{dl} (mA/mF)**
Cu/C-cavity	18.14	1.50	12.09
Cu/C-solid	8.68	1.30	6.68
Cu/C-fragment	8.25	0.71	11.62

Table S2. The C_{2+} partial current density(j_{C2+}) normalised to Cu content of the catalysts.

**Electrocatalyst**	**j_{C2+} (mA cm^{-2})**	**Cu content (wt%)**	**j_{C2+} normalised to Cu content (mA cm^{-2}/wt)**
Cu/C-cavity	18.14	30.09	60.47
Cu/C-solid	8.68	39.18	22.15
Cu/C-fragment	8.25	31.26	26.39

Table S3. Comparison of the results of typical nanoreactor electrocatalysts.

**Electrocatalyst**	**FE (C_{2+}) /%**	**Stability tested (h)**	**Refs.**
Cu/C-cavity	80.5% (H cell)	20	This work
	75.2 %(flow cell)	18	
Cu mesopore electrodes	46%	4	[6]
cavity Cu	75.6 ± 1.8%	12	[7]
multihollow Cu_2O	75.2 ± 2.7%	3	[8]
4.4-shell Cu	80%	11	[9]
3-shell HoMSs	77.0 ± 0.3%	8.3	[10]
$(Cu_2O@)_2Cu_2O$	22.22 ± 0.38% (CO to propanol)	5	[11]
cavity II Cu catalyst	21 ± 1% (CO to propanol)	2.5	[12]

References

[1] E. D. Snijder; M. J. M. te Riele; G. F. Versteeg; W. P. M. van Swaaij; *J. Chem. Eng. Data* **1995**, 40, 37-39.

[2] D. Wise; G. J. C. E. S; *Chem. Eng. Sci*. **1968**, 23, 1211-1216.

[3] A. A. UNVER and D. M. HIMMELBLAU; *J. Chem. Eng. Data* **1964**, 9,3.

[4] G. Kresse; D. Joubert; *Physical Review B,* **1999**, 59, 1758-177.

[5] J. P. Perdew; K. Burke; M. Ernzerhof; *Physical Review Letters,* **1996**, 77, 3865-3868.

[6] K. D. Yang; W. R. Ko; J. H. Lee; S. J. Kim; H. Lee; M. H. Lee and K. T. Nam; *Angew. Chem. Int. Ed.* **2017**, 56, 796-800.

[7] L. X. Liu; Y. Cai; H. Du; X. Lu; X. Li; F. Liu; J. Fu and J. J. Zhu; *ACS Appl. Mater. Interfaces,* **2023**, 15, 16673-16679.

[8] P.-P. Yang; X.-L. Zhang; F.-Y. Gao; Y.-R. Zheng; Z.-Z. Niu; X. Yu; R. Liu; Z.-Z. Wu; S. Qin; L.-P. Chi; Y. Duan; T. Ma; X.-S. Zheng; J.-F. Zhu; H.-J. Wang; M.-R. Gao and S.-H. Yu; *J. Am. Chem. Soc.* **2020**, 142, 6400-6408.

[9] Y. Xiao; M. Wang; H. Yang; H. Qiu; H. Lu; Y. Da; G. Chen; T. Jiang; W. Fu; B. Hu; J. Chen; L. Chen; Y. Ding; B. Cui; C. Jiang; Z. Sun; Y. Long; H. Yang; Z. Tian; L. Wang and W. Chen; *Adv. Energy Mater.* **2023**, 2302556

[10] C. Liu; M. Zhang; J. Li; W. Xue; T. Zheng; C. Xia and J. Zeng; *Angew. Chem. Int. Ed.* **2022**, 61, e202113498.

[11] H. Du; L.-X. Liu; P. Li; Q. Min; S. Guo and W. Zhu. *ACS Nano*, **2023**, 17, 8663-8670.

[12] T. T. Zhuang; Y. Pang; Z. Q. Liang; Z. Wang; Y. Li; C.S. Tan; J. Li; C. T. Dinh; P. De Luna; P. L. Hsieh; T. Burdyny; H. H. Li; M. Liu; Y. Wang; F. Li; A. Proppe; A. Johnston; D. H. Nam; Z. Y. Wu; Y. R. Zheng; A. H. Ip; H. Tan; L. J. Chen; S. H. Yu; S. O. Kelley; D. Sinton and E. H. Sargent; *Nat. Catal.* **2018**, 1, 946-951.
